# Novel phosphate-solubilizing bacteria enhance soil phosphorus cycling following ecological restoration of land degraded by mining

**DOI:** 10.1038/s41396-020-0632-4

**Published:** 2020-03-23

**Authors:** Jie-Liang Liang, Jun Liu, Pu Jia, Tao-tao Yang, Qing-wei Zeng, Sheng-chang Zhang, Bin Liao, Wen-sheng Shu, Jin-tian Li

**Affiliations:** 1 Institute of Ecological Science and Guangdong Provincial Key Laboratory of Biotechnology for Plant Development, School of Life Sciences, South China Normal University, Guangzhou, 510631 PR China; 2 School of Life Sciences, Sun Yat-sen University, Guangzhou, 510275 PR China

**Keywords:** Microbial ecology, Soil microbiology

## Abstract

Little is known about the changes in soil microbial phosphorus (P) cycling potential during terrestrial ecosystem management and restoration, although much research aims to enhance soil P cycling. Here, we used metagenomic sequencing to analyse 18 soil microbial communities at a P-deficient degraded mine site in southern China where ecological restoration was implemented using two soil ameliorants and eight plant species. Our results show that the relative abundances of key genes governing soil microbial P-cycling potential were higher at the restored site than at the unrestored site, indicating enhancement of soil P cycling following restoration. The *gcd* gene, encoding an enzyme that mediates inorganic P solubilization, was predominant across soil samples and was a major determinant of bioavailable soil P. We reconstructed 39 near-complete bacterial genomes harboring *gcd*, which represented diverse novel phosphate-solubilizing microbial taxa. Strong correlations were found between the relative abundance of these genomes and bioavailable soil P, suggesting their contributions to the enhancement of soil P cycling. Moreover, 84 mobile genetic elements were detected in the scaffolds containing *gcd* in the 39 genomes, providing evidence for the role of phage-related horizontal gene transfer in assisting soil microbes to acquire new metabolic potential related to P cycling.

## Introduction

Phosphorus (P) has long been considered the second most limiting nutrient for plant growth in terrestrial ecosystems after nitrogen (N) [1, 2]. In contrast to this conventional view, there is mounting evidence that P limitation could be as impactful as N limitation in terrestrial ecosystems [3, 4]. For instance, a global meta-analysis of 173 terrestrial studies revealed that the plant responses in terrestrial ecosystems to P addition were not significantly different from those to N addition [3]. Despite this, substantial variations in plant responses to P and/or N addition were found between sub-habitats (e.g., forest, grassland, tundra, and wetland) within terrestrial environments [3], indicating that whether the soil is more limited to P or N is dependent on the specific ecosystem considered. Nonetheless, although the relative magnitude of P and N limitation still remains open to debate [5, 6], there is little doubt that P limitation is widespread in terrestrial ecosystems [7]. Further, another global meta-analysis of 50 terrestrial studies showed that the plant responses in terrestrial ecosystems to P addition were more pronounced under elevated than under ambient N, indicating that P limitation in terrestrial ecosystems will become more pronounced under increasing atmospheric N deposition in the future [8]. Therefore, mitigating terrestrial P limitation is increasingly recognized as a major priority in ecosystem management and restoration [5, 9].

Heavily degraded ecosystems, such as abandoned mined lands, are characterized by extremely low levels of soil nutrients, including P [10–12]. Restoring these ecosystems requires the recovery of soil P cycling [12, 13]. Moreover, a thorough understanding of soil P cycling during the ecological restoration of such ecosystems will inform efforts to mitigate P limitation in terrestrial ecosystems [5, 9]. However, most studies on soil nutrient cycling during the restoration of heavily degraded mined lands have focused almost exclusively on N [14–16], leaving the impacts of ecological restoration on soil P cycling largely unknown. More surprisingly, even in the wider context of degraded lands, little attention has been paid to soil P cycling following such restoration efforts. Interestingly, there is evidence that soil P cycling in a re-established forest on abandoned, degraded agricultural land had not recovered even after over 50 years of reforestation [17], although the mechanisms underlying such a pattern are poorly studied.

Microbes play an integral role in soil P cycling, as they mediate bioavailable soil P [18–20]. Due to its high reactivity, P in soils can exist in many inorganic and organic forms that are recalcitrant to plant uptake [21]. In comparison with plants, microbes seem to harbor more diverse metabolic capacities to improve the bioaccessibility of various recalcitrant P forms in soils [18–20]. On one hand, a set of microbe-derived enzymes, such as acid phosphatase (encoded by *olpA*), alkaline phosphatase (*phoD*), phytase (*appA*), phosphonatase (*phnX*), and C-P lyase (*phnJ*), are able to release free orthophosphate from recalcitrant organic P forms [18–20]. On the other hand, a variety of organic acids, including citric acid, formic acid, gluconic acid, malic acid, and oxalic acid, are involved in the microbial solubilization of recalcitrant inorganic P forms [18–20, 22]. Among these organic acids, gluconic acid ranks as the most important in this process and has been studied widely [18, 20]. It has long been recognized that glucose dehydrogenase (GCD), a major enzyme responsible for the production of gluconic acid, is encoded by *gcd* [22, 23], while little is known about the genetic basis of other organic acids involved in the microbial solubilization of recalcitrant inorganic P [18, 20]. Nonetheless, exploring the genetic potential of soil microbes in solubilizing recalcitrant P forms is an important step towards a better understanding of the fundamental mechanisms driving soil P cycling [12, 13, 24]. In fact, there is emerging evidence that the aforementioned genes are widespread in various terrestrial environments, including grassland, forest, and cropped and fallow lands [25–27]. Of all these genes, *gcd* has frequently found to be predominant [25, 26].

Recent advances in metagenomics have not only allowed the simultaneous examination of multiple genes involved in a soil nutrient cycling process of interest but have also opened up the possibility of obtaining genomes of novel uncultivated microbial species that are of significance for soil functioning [24, 28]. To shed light onto the genetic mechanisms by which soil microbes can govern soil P cycling during ecological restoration, we used genome-centric metagenomics to explore how and why soil microbial P-cycling potential changes during the ecological restoration of a heavily degraded mined land deficient in P. Due to the lack of developed soil in the degraded land [12], its ecological restoration is essential to mimic and accelerate the natural processes of primary succession [10, 13]. This means that in the early stages of ecological restoration, a large proportion of soil P is likely bound to soil minerals (i.e., there is a predominance of recalcitrant inorganic P). We therefore hypothesized that the microbial genetic potential for soil P cycling (especially P solubilization) in the early stages of ecological restoration is predominantly represented by the *gcd* gene, which encodes a major enzyme involved in the microbial solubilization of recalcitrant inorganic P forms.

## Materials and methods

### Site description and field experiment

This study was conducted in a heavily degraded mined area (i.e., a mine tailings pond) located ~22 km southwest of Jiujiang city (29°40′52″N, 115°49′21″E), Jiangxi Province, China. The land has been abandoned for more than eight years, and the soil remains very acidic (pH 2.5, Table 1). As previously reported, an ecological restoration program was implemented in an area of ~4000 m^2^ located at the center of the degraded land [29]. Briefly, the target area for revegetation was divided into 20 plots, each 5 × 40 m, with 1-m walkways between them. Soil ameliorants (lime: 20 t ha^−1^; chicken manure: 40 t ha^−1^) were added to each plot at a depth of 10 cm to improve substrate conditions for plant growth. The concentrations of total and bioavailable P in chicken manure were 18.5 and 1.03 g kg^−1^ (on a dry weight basis), respectively. A mixture of eight plant species (including *Artemisia capillaris*, *Boehmeria nivea*, *Cynodon dactylon*, *Festuca arundinacea*, *Panicum repens*, *Paspalum notatum*, *Robinia pseudoacacia*, and *Sesbania cannabina*) was grown on the amended tailings from seeds or seedlings.

**Table 1:** Selected physico-chemical properties of the three types of soil samples collected at 3 and 4 years after ecological restoration initiation.

	Three years			Four years		
	UT^a^	ULRT	ALRT	UT	ULRT	ALRT
pH	2.87 ± 0.05 b^c^	7.13 ± 0.36 a	7.12 ± 0.21 a	2.64 ± 0.10 b	6.42 ± 1.17 a	7.29 ± 0.14 a
Eh (mV)	624 ± 13 a	347 ± 75 b	265 ± 8 b	618 ± 25 a	386 ± 144 b	174 ± 10 c
EC (mS m^−1^)	0.81 ± 0.07 a	0.62 ± 0.16 ab	0.36 ± 0.02 b	0.87 ± 0.21 a	0.50 ± 0.06 b	0.37 ± 0.01 b
NAG (kg H_2_SO_4_ t^−1^)	21.1 ± 2.48 a	0.8 ± 0.92 b	0.00 ± 0.00 b	29.2 ± 1.02 a	2.17 ± 1.07 b	2.20 ± 3.14 b
NAG-pH	2.66 ± 0.02 b	6.79 ± 1.26 a	7.84 ± 0.73 a	2.58 ± 0.05 b	5.81 ± 0.38 a	6.56 ± 0.59 a
Total C (g kg^−1^)	1.01 ± 0.04 b	3.26 ± 1.71 b	91.1 ± 13.5 a	1.19 ± 0.21 b	3.10 ± 0.11 b	68.7 ± 16.2 a
TOC^b^ (g kg^−^^1^)	0.22 ± 0.01 b	2.08 ± 0.99 b	63.0 ± 20.7 a	0.52 ± 0.22 b	1.36 ± 0.12 b	41.2 ± 14.5 a
WSOC (mg kg^−^^1^)	19 ± 2 b	113 ± 31b	1040 ± 257 a	54 ± 30 b	89 ± 35 b	569 ± 102 a
Total N (mg kg^−^^1^)	157 ± 6 b	347 ± 155 b	8090 ± 1705 a	207 ± 32 b	367 ± 31 b	6337 ± 1500 a
NH_4_^+^–N (mg kg^−^^1^)	9.3 ± 0.4 b	11.3 ± 3.9 ab	48.6 ± 32.7 a	5.6 ± 2.5 c	11.4 ± 3.9 b	18.6 ± 0.6 a
NO_3_^−^–N (mg kg^−^^1^)	0.13 ± 0.11 c	0.89 ± 0.25 b	1.34 ± 0.15 a	0.12 ± 0.21 b	6.28 ± 4.88 a	6.71 ± 0.20 a
Total P (mg kg^−^^1^)	49 ± 47 b	204 ± 43 b	1445 ± 441 a	90 ± 69 b	339 ± 83 b	983 ± 495 a
Bioavailable P (mg kg^−^^1^)	0.0 ± 0.0 b	14.9 ± 13.2 b	93.2 ± 46.9 a	0.5 ± 0.9 b	40.0 ± 33.6 a	44.4 ± 6.0 a
C/N	7.52 ± 0.09 c	10.8 ± 0.46 b	13.3 ± 0.54 a	6.76 ± 0.44 c	9.91 ± 0.62 b	12.6 ± 0.01 a
C/P	39.5 ± 12.4 b	39.6 ± 7.41 b	171 ± 24.3 a	22.8 ± 4.09 b	24.5 ± 3.30 b	216 ± 72.9 a
N/P	5.28 ± 1.61 b	3.64 ± 0.52 b	12.9 ± 1.74 a	3.48 ± 0.29 b	2.49 ± 0.39 b	17.1 ± 5.78 a
Moisture content (%)	10.6 ± 1.9 b	11.0 ± 4.6 b	34.0 ± 1.7 a	12.4 ± 3.4 b	14.1 ± 3.0 b	32.5 ± 3.1 a

C/N, C/P, and N/P referred to the mole ratios among total C, total N, and total P.

^a^UT the unreclaimed tailings, ULRT the unamended layer of the reclaimed tailings, ALRT the amended layer of the reclaimed tailings.

^b^TOC total organic carbon, WSOC water soluble organic carbon.

^c^Data (*n*  =  3; mean  ±  S.E.) for different types of samples with different letters were significantly different from each other (*P*  <  0.05, LSD).

### Sample collection

Sampling was conducted in July 2016 and 2017, representing 3 and 4 years after restoration treatment implementation, respectively. On each sampling date, three reclaimed plots were randomly selected for sampling. In each plot, one soil sample was collected from the amended layer of the reclaimed tailings (0–10 cm, ALRT) and from the unamended layer of the reclaimed tailings (11–20 cm, ULRT). One soil sample was also collected from the unreclaimed tailings (UT) next to each of the three sampled plots at a depth of 0–10 cm as controls. A major reason for the inclusion of ULRT in this study was that it provided us with an opportunity to obtain some clues for understanding the microbial mechanisms driving soil P cycling beyond the time frame of this study. This speculation was based on the observation that the plant roots were distributed mainly in ALRT on both sampling dates, but they are likely to go deeper into ULRT as time progresses [12]. In total, we collected 18 soil samples, as we considered three soil types (i.e., ALRT, ULRT, and UT), two sampling dates and three replicates for each soil type on each sampling date. The total area covered by our samples was ~144 m^2^, given that each of the 18 samples was comprised of three subsamples and each subsample was collected from three randomly distributed points covering an area of ~4 m^2^ but that the subsamples for ALRT were from the same points as those for ULRT (in different layers). All samples were kept under refrigeration until arrival at our laboratory, where they were stored at −20 °C until further processing.

### Physico-chemical analysis

Total and bioavailable soil P were determined according to the Murphy–Riley method [30] and Olsen’s method [31], respectively. A detailed description of the analytical methods used for the other selected soil physico-chemical properties, including the moisture content, redox potential (Eh), pH, net acid generation capacity (NAG), NAG-pH, and concentrations of ferric and ferrous iron, sulfate, total carbon, total organic carbon (TOC), water soluble organic carbon (WSOC), total N, ammonium nitrogen (NH_4_^+^–N), nitrate nitrogen (NO_3_^−^–N), and total and diethylenetriaminepentaacetic acid (DTPA)-extractable heavy metals, was previously published [29].

### DNA extraction, DNA sequencing, and data processing

The metagenomic DNA in our soil samples was extracted according to a previously described method [32], and the DNA quality was determined with a NanoDrop 2000 spectrophotometer (Thermo Scientific, USA). Each DNA sample was purified using silica-based columns and then used to construct a shotgun library (~500 bp average insert size), which was sequenced (150 or 250 bp paired-end reads; Table S1 in Supporting Information) using an Illumina MiSeq sequencer (Illumina, USA). The metagenomic datasets have been deposited at EMBL under accession number PRJEB31441.

### Metagenomics analysis

Sequencing reads were filtered by quality using our in-home Perl scripts as previously described [33], which included eliminating duplicated reads, removing reads with ≥5 “N” and filtering low-quality reads (quality score ≥ 30). High-quality (HQ) sequencing data from each sample were individually assembled into contigs using SPAdes v3.9.0 with various k-mer sizes (ranging from 31 to 121). The assemblies (≥500 bp) were retained for further analyses. Gene prediction was performed using Prodigal v2.6.3 [34]. The putative protein-coding sequences were compared (*e*-value threshold, 10^−5^) against databases including NCBI-nr, the extended COG, and KEGG to obtain their functional annotation using Diamond v0.9.24.125 [35]. Gene coverage was calculated using BBMap v36.x with the parameters *k* = 14, minid = 0.97, and build = 1. The relative abundance of a given gene functional category against the particular databases (i.e., NCBI-nr, COG, and KEGG) was calculated based on the gene coverage result using our in-home Perl scripts.

### Genome binning, taxonomic classification, and functional annotation

Scaffolds with a length <2000 bp in each metagenome assembly were removed from further analysis. HQ reads from each sample were separately mapped to each assembly to compute the coverage information of the scaffolds using BBMap with the abovementioned parameters. The scaffolds from each sample were individually binned using MetaBAT [36] with default parameters, taking into account both tetranucleotide frequencies and coverage information of the scaffolds. All bins were subjected to RefineM v0.0.14 [37] and then to manual examination for further refinement. The ‘lineage_wf’ pipeline in CheckM v1.0.4 [38] was used to evaluate contamination and the completeness of the genome bins through the identification and quantification of single-copy marker genes. Genomes were first dereplicated using dRep v1.4.3 [39] with default parameters, and those estimated to be more than 90% complete and less than 5% contaminated were selected for further analysis.

The taxonomic classifications of the selected genome bins were inferred from two phylogenetic trees constructed with the reference genomes using GTDB-Tk v2.1.15 [40] and PhyloPhlAn [41]. The phylogenetic tree was visualized using the Interactive Tree of Life online interface [42]. Gene calling was performed for individual genome bins using Prodigal [41], and protein-coding genes were assigned to the NCBI-nr, eggNOG, and KEGG databases for functional annotation using Diamond with an *e*-value threshold of 10^−5^.

### Identification and analysis of *gcd* genes in genome bins

Putative *gcd* genes from our genome bins were identified by KEGG annotation hits to K00170 (KEGG Orthology entry, which is the main identifier of quinoprotein GCD [EC:1.1.5.2]) and were further confirmed using InterProScan [43]. The public GCD sequences from the same phyla as those represented by our *gcd*-containing genome bins were downloaded from NCBI GenBank for phylogenetic analysis, including sequences that have been functionally verified [44]. Since the GCDs recovered in our study were all membrane bound, two protein sequences of the soluble GCD were also selected as the outgroup sequences. The GCD sequences were aligned using MUSCLE with default parameters [45]. All alignments were filtered by TrimAL with the parameters -gt = 0.95 and -cons = 50 to remove all columns with >95% gaps and all taxa with < 50% of the expected alignment columns [46]. The GCD tree was constructed using maximum likelihood with RAxML v8.0.26 [47] implemented by the CIPRES Science Gateway [48], with the parameters set as -f a -m PROTGAMMAJTT -# 100 -p 12345 -x 12345. The GCD tree was visualized and formatted in the Interactive Tree of Life online interface [42] using the Newick file with the best tree topology.

The 16S rRNA gene sequences of our *gcd*-containing genome bins were identified using RNAmmer [49] and were used to search for closely related 16S rRNA gene sequences in NCBI GenBank using BLASTn (*e*-value threshold, 10^−5^). The relative abundances of these genome bins were calculated as previously described [50]. Briefly, the HQ reads from each sample were mapped to all of the dereplicated genome bins with BBMap as described above. The coverage of each genome bin was calculated as the average scaffold coverage, and each scaffold was weighed by its length in base pairs. Then, the relative abundance of each genome bin in each sample was calculated as its coverage divided by the total coverage of all genome bins in each sample.

### Construction of trees to infer horizontal gene transfer (HGT)

Together with the *gcd*-containing bins recovered in this study, 44 other *gcd*-harbouring genomes affiliated with the same phyla as those covered by our bins were downloaded from NCBI GenBank for the construction of a genome-based phylogenetic tree using PhyloPhlAn [41]. Two additional genomes from Euryarchaeota were selected as the outgroup. The 400 concatenated universal proteins identified by PhyloPhlAn [41] were also used to construct the phylogenetic tree by RAxML v8.0.26 [47], with the parameters set as -f a -m PROTGAMMAJTT -# 100 -p 12345 -x 12345. The Newick file with the best tree topology was uploaded to the Interactive Tree of Life online interface [42] for visualization and formatting. The GCD proteins encoded by the genomes used for the construction of the phylogenetic trees were identified. Then, the relevant protein sequences were aligned and used for GCD tree construction as described above.

### Statistical analysis

The least significant difference (LSD) test was used to identify the differences in physico-chemical characteristics among the three soil sample types as well as functional categories/genes at a 0.05 significance level. Pearson correlation analysis and Spearman correlation analysis were used to correlate the concentrations of total and bioavailable P with the relative abundances of P-related genes and *gcd*-harbouring genome bins via the vegan package within the R statistical computing environment [51]. A random forest analysis [52] was performed to identify which microbial genes involved in soil P cycling were the most important determinants of bioavailable soil P. This analysis is different from traditional classification and regression tree analysis, as only approximately two-thirds of the data were used to construct a collection of classification trees, and the remaining data (out-of-bag, OOB) were used to assess the fit of each tree (the prediction error, i.e., OOB error). The variables included in the model leading to the smallest OBB error were selected as important predictors [53]. To evaluate the importance of each predictor, the increase in the mean square error between observations and OOB predictions was calculated after randomly permuting the data for that predictor [52].

## Results

### Increased levels of total and bioavailable soil P

The concentrations of total P in our soil samples on the two sampling dates were in the order of ALRT > ULRT > UT (Table 1), corresponding to the degree to which the three types of samples were influenced by the restoration measures. Significant differences (*P* < 0.05) were found between ALRT and the other two types of soil samples but not between ULRT and UT (Table 1), which could be attributed at least partly to the large variations within the UT samples. A similar pattern was observed for bioavailable soil P (Table 1). In addition, soil pH, the levels of the other two major nutrient elements (i.e., C and N) in the soil, and the ratios between C, N, and P were shown to be significantly (*P* < 0.05) higher in ALRT than in UT (Table 1). Note that the soil pH and NAG-pH in ULRT were comparable to (*P* > 0.05) those in ALRT (Table 1), indicating that the restoration measures had a positive effect at a lower depth than in the amended layer. A possible explanation for this pattern is that the restoration measures led to a low-oxygen condition in ULRT (as indicated by a lower Eh compared with UT, Table 1) and thus limited the proliferation of microbes driving tailings acidification [29]. Few significant (*P* < 0.05) differences in soil physico-chemical properties were observed between the years for the same sample type (statistical results not shown).

### Enhanced soil microbial P-cycling potential

We analysed ~283 million short-read sequences from the 18 metagenomes obtained in this study and identified 509,265 to 4,732,804 putative protein-coding genes in individual samples. Approximately 58–69% of the genes could be assigned to KEGG or COG catalogs. To explore the changes in soil microbial P-cycling potential, further analyses focused exclusively on genes encoding proteins that are involved in the microbial turnover of soil P (Table S2) [25].

**Fig. 1:**
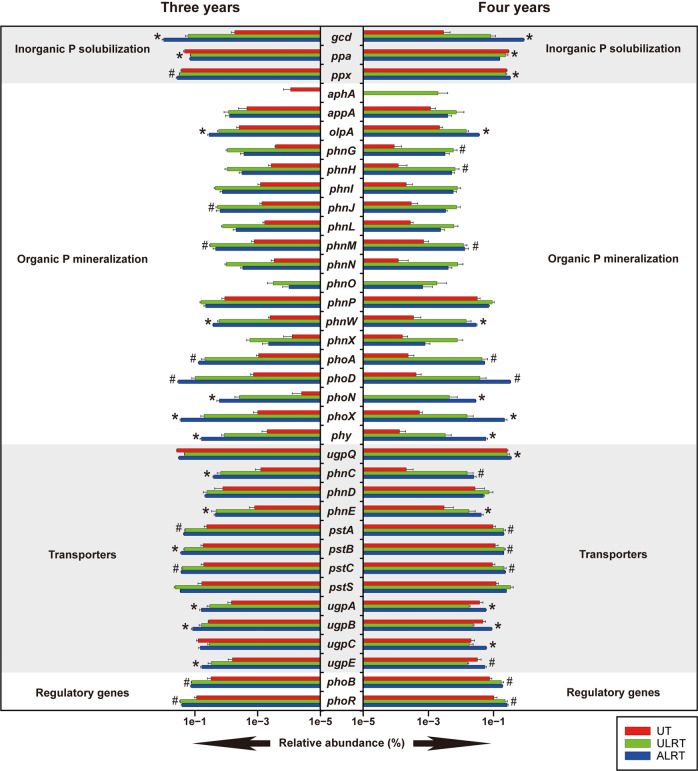
The effects of ecological restoration on the relative abundances of genes responsible for soil microbial P-cycling potential. Data (*n* = 3; mean ± S.E.) of soil samples collected at 3 and 4 years after the initiation of the ecological restoration were presented in the left and right panel, respectively. UT, ALRT, and ULRT represent soil samples collected from unrestored mine tailings, the amended layer (0–10 cm) of the restored mine tailings and the unamended layer (11–20 cm) of the restored mine tailings, respectively. Asterisk symbol indicates significant (*P* < 0.05, LSD) differences between UT and ALRT (i.e. ALRT > UT). Hash symbol indicates significant differences between the three types of soil samples (i.e. ALRT > ULRT > UT).

A total of 36 genes involved in the microbial turnover of soil P were detected in our metagenomes (Fig. 1). They could be roughly divided into the three following subgroups: solubilization (including inorganic P solubilization and organic P mineralization), transporter, and regulatory genes [26]. The majority (~70%) of these genes (including *gcd*, *phoD*, and *phoA*) were found in a higher abundance in ALRT than in UT and ULRT (*P* < 0.05, Fig. 1), indicating that the soil microbial P-cycling potential of the heavily degraded land was considerably enhanced by the restoration measures. In this regard, the *gcd* gene was a striking example for two reasons. First, *gcd* was among the most predominant genes responsible for soil microbial P cycling (especially P solubilization) in the degraded land, supporting our hypothesis regarding its predominance. Second, the relative abundance of *gcd* was 183 and 289 times higher in ALRT than in UT on the two sampling dates, respectively (Fig. 1). Notably, the relative abundances of several genes encoding C-P lyase (e.g., *phnG*, *phnH*, and *phnI*) in ULRT were comparable with or higher than those in ALRT (Fig. 1), indicating that the restoration measures improved the genetic potential of microbial C-P lyase not only in ALRT but also in ULRT [18, 19].

### Linkages between soil microbial P-cycling potential and soil P status

**Fig. 2:**
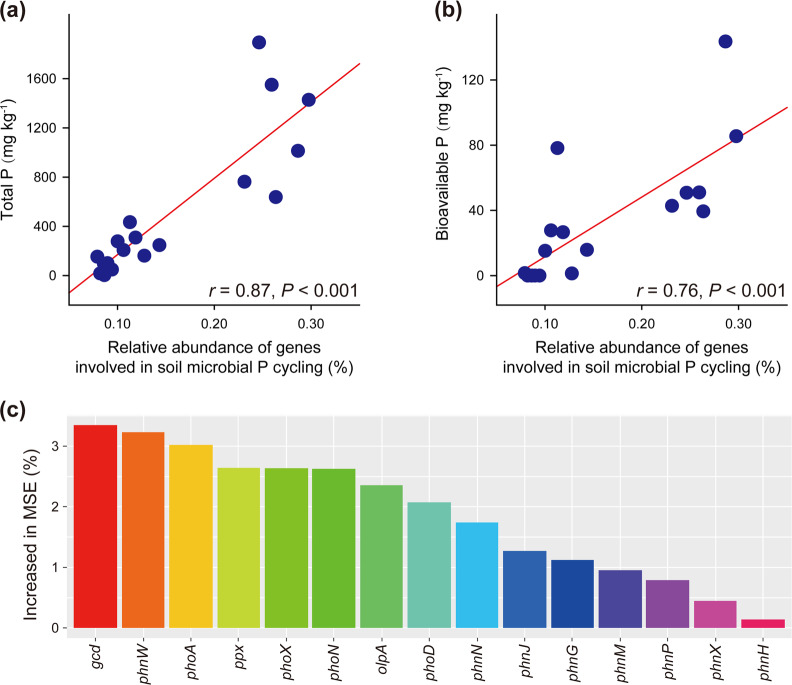
The linkages between genes responsible for soil microbial P-cycling potential and soil P status. Relationships between the relative abundance of all genes involved in soil microbial P cycling and total soil P (**a**) or bioavailable soil P (**b**) are shown. Panel (**c**) shows the significant (*P* < 0.05) gene predictors of bioavailable soil P, identified by random forest analysis. The data of the two sampling dates were analyzed together.

There was a positive relationship between the relative abundance of all genes involved in the microbial turnover of soil P and the concentration of total soil P (*r* = 0.87, *P* < 0.001; Fig. 2a), which suggests that the addition of P-rich chicken manure to the degraded land stimulated its soil microbial P-cycling potential. This speculation was made because lime (the other soil amendment used in this study) tends to cause P precipitation and has been frequently reported to decrease soil P cycling [54]. Furthermore, the relative abundance of all genes involved in the microbial turnover of soil P was also positively correlated with the concentration of bioavailable soil P (*r* = 0.76, *P* < 0.001; Fig. 2b), indicating an important role of the enhanced soil microbial P-cycling potential in the accumulation of bioavailable soil P. The random forest analysis showed that 15 of the 36 P-related genes could be considered determinants of the concentration of bioavailable soil P (Fig. 2c). Remarkably, among them, *gcd* was the most important (Fig. 2c). In addition, Pearson correlation analysis showed significant positive correlations between the 15 genes and bioavailable soil P (*P* < 0.05, Table S3).

### Novel genome bins driving the enhanced soil microbial P cycling potential

**Fig. 3:**
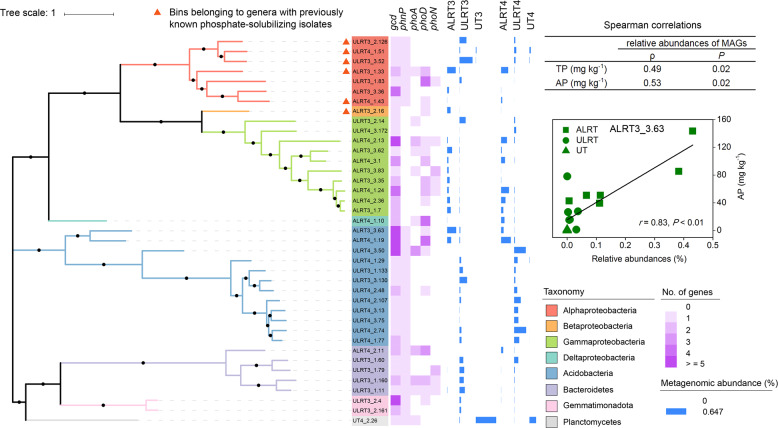
Analysis of the 39 high-quality metagenome-assembled genomes harboring sequences of *gcd* genes. The left panel shows the maximum-likelihood phylogenetic tree constructed using PhyloPhlAn. Bootstrap values were based on 100 replicates, and percentages higher than 70% are shown with black circles. Numbers of genes encoding enzymes responsible for P solubilization identified in the genomes are shown in the middle panel. *gcd* encoding quinoprotein glucose dehydrogenase, *phnP* C-P lyase subunit (PhnP), *phoA* alkaline phosphatase (PhoA), *phoD* alkaline phosphatase (PhoD), and *phoN* acid phosphatase (class A). Spearman correlations between the relative abundance of the 39 genomes and concentrations of total soil P (TP) or bioavailable soil P (AP) are listed in the table present in the right part of the figure. MAGs metagenome-assembled genomes. The relationship between the relative abundance of the genome bin ALRT3_3.63 and AP concentration is shown in the inset present in the right part of the figure.

The assembly and binning processes generated 424 microbial genome bins with more than 90% completeness and less than 5% contamination (near-complete genomes, Table S4). Among them, 39 contained *gcd* genes (Fig. 3 and Table S5). These genomes represented a variety of bacteria in the Acidobacteria phylum (12 of the 39 genomes), α-Proteobacteria class (7), β-Proteobacteria class (1), γ-Proteobacteria class (10), δ-Proteobacteria class (1), Bacteroidetes phylum (5), Gemmatimonadaceae family (2), and Isosphaeraceae family (1). Remarkably, only six (i.e., ~15%) of the 39 genomes belonged to microbial genera with previously known phosphate-solubilizing isolates (Fig. 3 and Table S5) [20], indicating that the other 33 genomes represent a repertoire of novel phosphate-solubilizing microbial taxa.

The 39 genomes differed considerably from one another in the number of copies of the *gcd* gene, ranging from 1 to 11 (Fig. 3 and Table S5). None of the six genomes belonging to the microbial genera with previously known phosphate-solubilizing isolates contained more than two copies of the *gcd* gene. In contrast, 5 of the 33 genomes representing novel phosphate-solubilizing microbial taxa (e.g., bin ALRT3_3.63 and ALRT4_1.19) were found to harbor greater than five *gcd* genes individually (Fig. 3 and Table S5). In addition, the 33 novel genomes also tended to have more genes (e.g., *phoD*) involved in organic P mineralization than the other six genomes (Fig. 3).

The relative abundance of these 39 individual genomes varied greatly across sample types (Fig. 3). However, a large majority of the genomes that occurred at a relatively high abundance were those representing novel phosphate-solubilizing microbial taxa (e.g., bin ALRT3_3.63 and bin UT4_2.26, Fig. 3). Spearman correlation analysis showed that the relative abundance of all 39 genomes was positively correlated with both the total (*ρ* = 0.49, *P* = 0.02) and bioavailable (*ρ* = 0.53, *P* = 0.02) P in soil (Fig. 3). Moreover, the relative abundance of five individual genomes (i.e., bin ALRT3_3.63, bin ALRT3_3.36, bin ALRT3_3.35, bin ALRT3_3.62, and bin ALRT3_3.83) was positively correlated with the concentration of bioavailable soil P (*r* ≥ 0.70, *P* < 0.01; Fig. 3 and Table S6), with bin ALRT3_3.63 being the only one having a correlation coefficient >0.80 with the concentration of bioavailable soil P (*r* = 0.83, *P* < 0.01). Remarkably, all five genomes belonged to genera without previously identified phosphate-solubilizing isolates (Fig. 3).

### Diversity and phylogeny of the genes encoding GCD in the genome bins

**Fig. 4:**
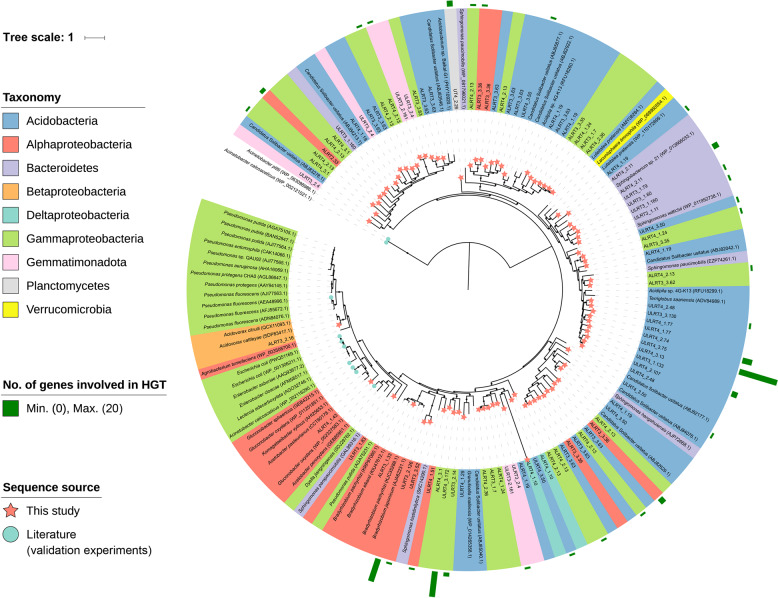
Phylogenic analysis of quinoprotein glucose dehydrogenases (GCDs). Bootstrap values were based on 100 replicates, and only bootstrap values higher than 70% are shown with black circles. The accession number of each published protein is shown in parenthesis. The new GCDs recovered in our study are indicated with red stars. The published GCDs with functional validation by experimentation in the literature are indicated with green circles. Numbers of genes annotated as mobile genetic elements involved in horizontal gene transfer (HGT) on the same scaffolds with *gcd* genes are indicated with green bars outside the phylogenetic tree. Details on mobile genetic elements involved in HGT were provided in Table 2 and Table S7.

In total, 89 genes encoding GCD were detected in the 39 *gcd*-containing genome bins (Fig. 4). Remarkably, the majority of these GCD proteins were unique from the published reference sequences and formed distinct clusters in the tree (Fig. 4). These results indicate the discovery of many potentially new *gcd* genes in this study. Remarkably, these putative new genes were widely distributed across a variety of taxa affiliated with Acidobacteria, Bacteroidetes, Gemmatimonadota, Planctomycetes, and Proteobacteria (Fig. 4). However, the GCD protein sequences from Proteobacteria clustered with those from the other three phyla, indicating the involvement of HGT during the acquisition of *gcd* in some of these bacteria (Fig. 4) [55]. This finding was supported by the comparison of a genome-based phylogenetic tree and the GCD protein tree, as a mismatching branching pattern was obvious in the two trees (Fig. S1). The comparison also indicated that some members of α-Proteobacteria, γ-Proteobacteria, Bacteroidetes, and Gemmatimonadota possibly acquired *gcd* genes through multiple independent HGT events [56].

### Mobile genetic elements in the scaffolds with the gcd genes in the genome bins

A total of 84 mobile genetic elements involved in HGT were found in the scaffolds with the *gcd* genes in the 39 genome bins (Table 2). They were distributed in 21 of the 39 genome bins, with their numbers varying from one to 20 in each bin (Fig. 4 and Table 2). Notably, 17 of the 21 genome bins belonged to the novel phosphate-solubilizing microbial taxa (Fig. 3 and Table 2). Phage-related elements, detected in nine genome bins (Table 2), constituted as much as 23% of the total mobile genetic elements, suggesting the involvement of phages in HGT [55]. In agreement with this, elements encoding integrases (markers of temperate phages) [57] were also detected in seven genome bins (Table 2).

**Table 2:** Summary of mobile genetic elements detected in the scaffolds containing *gcd* genes in the 39 genome bins reconstructed in this study.

Bins^a^	Scaffolds^b^	Total	Phage related	Integrases	Recombinases	Transposable	Others
ALRT4_1.19	Scaffold2.1	1	0	0	0	1 (100)	0
ULRT4_2.74	Scaffold7.1	5	1 (20)^c^	1 (20)	2 (40)	2 (40)	0
ULRT4_3.50	Scaffold9.1	1	0	0	0	1 (100)	0
ULRT4_3.50	Scaffold9.2	1	0	0	0	1 (100)	0
ULRT4_3.50	Scaffold9.3	1	0	0	0	1 (100)	0
ULRT4_3.50	Scaffold9.4	1	1 (100)	1 (100)	0	0	0
ULRT4_3.75	Scaffold10.1	20	5 (25)	5 (25)	10 (50)	2 (10)	0
ULRT3_1.133	Scaffold11.1	3	0	0	1 (33)	2 (67)	0
ULRT3_3.130	Scaffold12.1	1	0	0	1 (100)	0	0
ALRT3_1.33	Scaffold13.1	2	0	0	2 (100)	0	0
ALRT3_3.36	Scaffold14.1	1	0	0	1 (100)	0	0
ALRT3_3.36	Scaffold14.2	3	2 (67)	0	1 (33)	0	0
ULRT4_1.51	Scaffold16.1	1	0	0	1 (100)	0	0
ULRT3_2.126	Scaffold18.1	12	4 (33)	2 (17)	7 (58)	1 (8)	0
ULRT3_3.52	Scaffold19.1	1	0	0	1 (100)	0	0
ULRT3_1.11	Scaffold21.1	1	0	0	1 (100)	0	0
ULRT3_1.160	Scaffold22.1	1	1 (100)	0	0	0	0
ULRT3_1.79	Scaffold24.1	3	0	0	2 (67)	1 (33)	0
ALRT4_1.24	Scaffold31.1	1	0	0	1 (100)	0	0
ALRT4_2.13	Scaffold32.1	1	0	0	1 (100)	0	0
ALRT4_2.13	Scaffold32.2	1	0	0	1 (100)	0	0
ALRT4_2.13	Scaffold32.3	1	0	0	1 (100)	0	0
ALRT4_2.13	Scaffold32.4	1	1 (100)	0	0	0	0
ALRT4_3.1	Scaffold34.1	1	0	0	1 (100)	0	0
ULRT4_3.172	Scaffold35.1	13	3 (23)	2 (15)	6 (46)	4 (31)	0
ULRT3_2.14	Scaffold36.1	2	0	1 (50)	1 (50)	0	0
ULRT3_2.161	Scaffold37.1	1	0	0	1 (100)	0	0
UT4_2.26	Scaffold39.1	3	1 (33)	1 (33)	0	1 (33)	0

^a^The bins were named after the sample from which they were assembled and binned. For example, the name ‘ALRT4_1.19’ meant that this bin was generated from the metagenomic dataset of the sample ALRT4_1 (representing the first replicate sample collected from the amended layer of the reclaimed tailings at 4 years after ecological restoration initiation), as the 19th bin of the genome bins recovered in this study.

^b^Detailed functional annotations were provided in Table S7.

^c^Percentages of each type of element were given in parentheses.

## Discussion

As an ecological and evolutionary factor, P is thought to override all other elements, including C and N [7]. However, while genome-centric metagenomics has recently allowed great advances in understanding the important role of microbes lacking prior genomic characterization in soil C and N cycling [58, 59], few metagenome-assembled genomes encoding enzymes responsible for soil P cycling have been reported in the literature [60]. Here, we reconstructed 39 near-complete genomes involved in soil P cycling from 18 metagenomes associated with an ecological restoration project, which enabled us to deduce that certain previously unknown phosphate-solubilizing bacteria were the main drivers of the enhancement in soil P cycling following restoration. Moreover, we obtained evidence that soil microbes can shape new P-related metabolic potential via a phage-related HGT mechanism.

### The *gcd* gene was the best predictor of enhanced soil P cycling

The concentrations of total and bioavailable soil P in UT (Table 1) were very low compared with those reported for similar degraded lands [61–64]. Our restoration measures improved the soil P cycling in the degraded mined land, given that the concentrations of total and bioavailable soil P in ALRT were comparable with those in many arable soils [64, 65]. While the enhancement in total soil P could be attributed to the addition of chicken manure, the elevated bioavailability of soil P reflected the increase in soil microbial P-cycling potential (especially P solubilization) following the restoration measures [18, 19].

The number of genes responsible for soil microbial P-cycling potential observed in this study (Fig. 1) was comparable with those reported in similar metagenomic studies [25–27]. However, it was striking that the relative abundance of 26 (i.e., 72%) of the 36 P-related genes observed in this study was significantly enhanced by the restoration measures, given that fertilization, the soil P stock level and the land-use regime were previously shown to impact the relative abundance of only a small proportion of genes involved in soil P cycling [25–27]. More importantly, of these 26 genes, *gcd* was found to be the most predominant in the majority of our samples. This pattern was reasonable since it has been proposed that the microbial solubilization of inorganic P is a major process involved in soil P cycling in soils where inorganic P prevails [25]. Due to technical difficulties in quantifying the chemical species of soil P [66], direct information on the relative abundances of inorganic and organic P forms was not obtained in this study or in comparable previous studies [25, 26]. However, the predominance of inorganic P in our soil samples could be deduced from the low level of total C content in the soils (Table 1) [25].

Despite the possibility that soil microbial P-cycling potential (as indicated by the occurrence of the genes involved) does not necessarily reflect the actual activity of the soil enzymes responsible for soil P cycling [25], we found evidence that the relative abundance of all genes involved in soil microbial P cycling was positively related not only to the concentration of the total soil P but also to that of the bioavailable soil P (Fig. 2a, b). These results indicate the validity of metagenomics as an effective approach for exploring the role of microbes in soil P cycling [24]. It should be noted that the relative importance of individual genes in governing soil P cycling has not yet been addressed in the literature, although metagenomics has allowed one to do so. Interestingly, the relative abundance of the *gcd* gene was the most important determinant of the concentration of bioavailable soil P in the degraded land (Fig. 2c). There are at least two reasonable explanations for this finding. One is the predominance of *gcd* across our soil samples (Fig. 1), while the other is the putative predominance of inorganic P forms in the samples [66].

### Novel *gcd*-containing genome bins drove the enhancement of soil P cycling

Given that the known enzymes responsible for the microbial solubilization of recalcitrant soil P are encoded by a small number of genes [18, 19], the ability of microbes to solubilize P is considered to be poorly conserved (generally at a phylogenetic resolution finer than the genus level) [67]. As such, a thorough understanding of the microbial contribution to soil P cycling requires the identification of individual microbial species that have the potential to solubilize recalcitrant soil P. However, addressing the contribution of microbes to soil P cycling at the genus level is still common in recent metagenomic studies [for e.g., refs. 25, 26]. This could be explained largely by two reasons. First, the currently identified phosphate-solubilizing bacterial isolates are well represented by several genera [20]. Second, it is difficult to recover genomes from metagenomic data obtained from soil samples with high microbial diversity [59]. To the best of our knowledge, only two genome bins involved in microbial P solubilization (as indicated by the presence of genes encoding phytases) have been reported in the literature [60]. Despite this, the role of these two genome bins in soil P cycling remains unclear [60].

The 39 *gcd*-containing genome bins obtained in this study greatly expand the known diversity of phosphate-solubilizing microbes. As recently summarized [20], the currently known phosphate-solubilizing bacterial isolates are generally affiliated with 17 genera, which belong to three phyla (i.e., Actinobacteria, Firmicutes, and Proteobacteria). Interestingly, over half of our *gcd*-containing genome bins represented members of four other phyla (Acidobacteria, Bacteroidetes, Gemmatimonadota, and Planctomycetes; Fig. 3 and Table S5) that were not previously known to have phosphate-solubilizing isolates [20]. This means that our results more than doubled the phylum diversity of bacteria involved in inorganic P solubilization. Moreover, the strong correlations of the relative abundances of the five individual genome bins representing novel phosphate-solubilizing bacteria with bioavailable soil P (Fig. 3 and Table S6) indicated that they were among the major drivers of the enhancement in soil P cycling following restoration [25]. In this regard, the bin ALRT3_3.63, affiliated with Acidobacteria, was remarkable, as the correlation coefficient of its relative abundance with bioavailable soil P (*r* = 0.83, Fig. 3) was not only the highest among those of the five genome bins but also exceeded that of the relative abundance of all the genes involved in soil P cycling (*r* = 0.76, Fig. 2b). These results could be attributed at least partly to the high number of *gcd* genes in the bin ALRT3_3.63 (i.e., 11, Fig. 3) [25]. Notably, another genome affiliated with Acidobacteria was reported to harbor as many as 10 *gcd* genes [68], although the functions of these genes remain to be validated.

### HGT contributed to the acquisition of *gcd* in novel phosphate-solubilizing bins

In P-limited habitats such as the degraded mined land studied here, the ability of microbes to solubilize recalcitrant soil P is an important trait that determines their fitness in these habitats [69]. It is thus likely that to adapt to hostile edaphic conditions, some microbes must acquire new genes encoding enzymes responsible for solubilizing recalcitrant soil P [46, 69]. Little evidence for the role of HGT in mediating the ability of microbes to acquire new genes involved in the solubilization of inorganic P is currently available in the literature [70], although HGT has been widely recognized as a major strategy by which microbes acquire new genes [55]. By employing gene-centric metagenomics, Coleman and Chisholm [71] revealed that natural populations of the model marine microbial genus *Prochlorococcus* differed considerably in their P utilization genes (i.e., those involved in organic P mineralization and regulation). The authors further ascribed their findings to HGT that was putatively driven by P limitation, though their opinion was later thought to be dubious [72]. Intriguingly, our study provides evidence that HGT contributes to the microbial acquisition of new genes involved in the solubilization of inorganic P (Figs. 4 and S1). More importantly, we went beyond the existing work on the role of HGT in mediating the ability of microbes to acquire new genes involved in P cycling [e.g., ref. 71], as the phage-related mobile genetic elements observed in this study support a widely presumed but not yet reported phage-related HGT mechanism for this process (Fig. 4 and Table 2). However, we cannot exclude the possibility that mutation might also play an important role in the acquisition of the new *gcd* genes in some of the genome bins [55], as a considerable proportion of the 39 genomic bins contained potentially new *gcd* genes but lacked the mobile genetic elements involved in HGT (Fig. 4 and Table 2).

## Conclusions

We conclude that certain phosphate-solubilizing bacteria (as indicated by the occurrence of the *gcd* gene) from phyla that were not previously known to have isolates capable of solubilizing phosphate play an important role in driving the enhancement of soil P cycling following the restoration of a heavily degraded mined land. Furthermore, phage-related HGT is likely involved in the acquisition of *gcd* in some of these bacteria. Our findings may have important implications for understanding the microbial mechanisms underlying soil P cycling in many other terrestrial ecosystems, given that *gcd* has been reported to prevail in various types of soils [25–27].

## Supplementary information

Supporting information
