# Supplementary material for: Novel phosphate-solubilizing bacteria enhance soil phosphorus cycling following ecological restoration of land degraded by mining
Source: ISME J. 2020 Mar 23;14(6):1600–13. doi: 10.1038/s41396-020-0632-4 (PMC7242446; doi:10.1038/s41396-020-0632-4)
Supplement: Supplementary file 1 — Supporting information [file 41396_2020_632_MOESM1_ESM.docx]

**Table S1.** Statistics for sequencing data of the three types of soil samples collected at three and four years after ecological restoration initiation.

| **Sample No.** | **Total reads number** | | | | | | | Scaffolds (≥ 500 bp) | | | | |
| --- | --- | --- | --- | --- | --- | --- | --- | --- | --- | --- | --- | --- |
|  | **Raw reads** | **Raw bases** | **Ave. read len. (nt)** | **Trimmed reads** | **Trimmed bases** | **Ave. read len. (nt)** | **Clean ratio** | **Total No.** | **Ave. len. (bp)** | **Ave. GC (%)** | **N50 (bp)** | **Longest scaffold (bp)** |
| UT3_1^#^ | 1.30E+08 | 3.26E+10 | 250 | 1.30E+08 | 3.16E+10 | 244 | 0.990 | 252676 | 1763 | 54 | 3361 | 656367 |
| UT3_2 | 7.95E+07 | 1.99E+10 | 250 | 7.90E+07 | 1.93E+10 | 244 | 0.990 | 221490 | 1789 | 58 | 3498 | 478837 |
| UT3_3 | 1.01E+08 | 2.52E+10 | 250 | 1.00E+08 | 2.44E+10 | 244 | 0.990 | 263515 | 1745 | 57 | 3156 | 433747 |
| ULRT3_1 | 1.41E+08 | 2.11E+10 | 150 | 1.29E+08 | 1.88E+10 | 145 | 0.920 | 1029005 | 1199 | 61 | 1303 | 1434537 |
| ULRT3_2 | 8.46E+07 | 2.12E+10 | 251 | 8.44E+07 | 2.07E+10 | 245 | 1.000 | 1555199 | 1090 | 61 | 1082 | 1956948 |
| ULRT3_3 | 9.13E+07 | 2.28E+10 | 250 | 9.09E+07 | 2.23E+10 | 245 | 1.000 | 1178170 | 1099 | 60 | 1116 | 1036712 |
| ALRT3_1 | 5.52E+07 | 1.38E+10 | 250 | 5.50E+07 | 1.35E+10 | 245 | 1.000 | 916637 | 895 | 63 | 829 | 405184 |
| ALRT3_2 | 5.10E+07 | 1.28E+10 | 250 | 5.08E+07 | 1.25E+10 | 245 | 1.000 | 932133 | 895 | 65 | 832 | 202922 |
| ALRT3_3 | 8.46E+07 | 2.12E+10 | 251 | 8.45E+07 | 2.07E+10 | 245 | 1.000 | 3036850 | 914 | 65 | 859 | 1388860 |
| UT4_1 | 2.21E+08 | 3.32E+10 | 150 | 2.08E+08 | 3.05E+10 | 147 | 0.940 | 327959 | 1863 | 58 | 3170 | 471934 |
| UT4_2 | 2.28E+08 | 3.42E+10 | 150 | 2.16E+08 | 3.16E+10 | 146 | 0.950 | 299536 | 1860 | 58 | 3203 | 278697 |
| UT4_3 | 2.30E+08 | 3.45E+10 | 150 | 2.19E+08 | 3.20E+10 | 146 | 0.950 | 414104 | 1657 | 58 | 2602 | 672966 |
| ULRT4_1 | 2.10E+08 | 3.15E+10 | 150 | 1.99E+08 | 2.97E+10 | 149 | 0.950 | 305833 | 1912 | 60 | 4268 | 1622153 |
| ULRT4_2 | 2.37E+08 | 3.56E+10 | 150 | 2.25E+08 | 3.36E+10 | 149 | 0.950 | 828586 | 1483 | 57 | 2098 | 1574773 |
| ULRT4_3 | 2.02E+08 | 3.04E+10 | 150 | 1.92E+08 | 2.86E+10 | 149 | 0.950 | 904524 | 1383 | 61 | 1714 | 2629447 |
| ALRT4_1 | 2.37E+08 | 3.55E+10 | 150 | 2.27E+08 | 3.38E+10 | 149 | 0.960 | 1048577 | 970 | 64 | 950 | 401692 |
| ALRT4_2 | 2.24E+08 | 3.36E+10 | 150 | 2.14E+08 | 3.18E+10 | 149 | 0.950 | 852186 | 960 | 64 | 927 | 499011 |
| ALRT4_3 | 2.27E+08 | 3.41E+10 | 150 | 2.17E+08 | 3.24E+10 | 149 | 0.95 | 854528 | 932 | 65 | 881 | 701208 |

^#^UT, the unreclaimed tailings; ULRT, the unamended layer of the reclaimed tailings; ALRT, the amended layer of the reclaimed tailings. The numbers right after UT, ULRT or ALRT referred to the time when the samples were collected. The numbers right after underscores referred to one of the three replicates of the same sample type. For example, UT3_1 referred to the first replicate sample collected from the unreclaimed tailings at three years after ecological restoration initiation.

**Table S2.** All investigated genes related to microbial turnover of soil P with their KO numbers, COG numbers and the corresponding enzymes.

| **Gene** | **Description** | **KEGG ID** | **COG ID** |
| --- | --- | --- | --- |
| P solubilization | | | |
| *Inorganic P solublization* | | | |
| *gcd* | PQQGDH (quinoprotein glucose dehydrogenase) | K00117 | COG4993 |
| *ppa* | inorganic pyrophosphatase | K01507 | COG0221 |
| *ppx* | exopolyphosphatase | K01524 | COG0248 |
| *Organic P mineralization* | | | |
| *phoN* | acid phosphatase (class A) | K09474 | COG0671 |
| *aphA* | acid phosphatase (class B) | K03788 | COG3700 |
| *appA* | 4-phytase | K01093 | COG4247 |
| *olpA* | acid phosphatase (class C) | K01078 |  |
| *phnA* | phosphonoacetate hydrolase | K06193 | COG2824 |
| *phnF* | C−P lyase subunit (PhnF) | K02043 | COG2188 |
| *phnG* | C−P lyase subunit (PhnG) | K06166 | COG3624 |
| *phnH* | C−P lyase subunit (PhnH) | K06165 | COG3625 |
| *phnI* | C−P lyase subunit (PhnI) | K06164 | COG3626 |
| *phnJ* | C−P lyase subunit (PhnJ) | K06163 | COG3627 |
| *phnK* | C−P lyase subunit (PhnK) | K05781 | COG4107 |
| *phnL* | C−P lyase subunit (PhnL) | K05780 | COG4778 |
| *phnM* | C−P lyase subunit (PhnM) | K06162 | COG3454 |
| *phnN* | C−P lyase subunit (PhnN) | K05774 | COG3709 |
| *phnO* | C−P lyase subunit (PhnO) | K09994 | COG0454 |
| *phnP* | C−P lyase subunit (PhnP) | K06167 | COG1235 |
| *phnW* | 2-aminoethylphosphonate (AEP) - pyruvate transaminase | K03430 | COG0075 |
| *phnX* | phosphonatase | K05306 |  |
| *phoA* | alkaline phosphatase (PhoA) | K01077 | COG1785 |
| *phoD* | alkaline phosphatase (PhoD) | K01113 | COG3540 |
| *phoX* | alkaline phosphatase (PhoX) |  | COG3211 |
| *phy* | 3-phytase | K01083 |  |
| *opd* | phosphotriesterase | K07048 | COG1735 |
| *ugpQ* | glycerophosphoryl diester; phosphodiesterase | K01126 | COG0584 |
| P transportation | | | |
| *phnC* | phosphonate transporter subunit PhnC | K02041 | COG3638 |
| *phnD* | phosphonate transporter subunit PhnD | K02044 | COG3221 |
| *phnE* | phosphonate transporter subunit PhnE | K02042 | COG3639 |
| *pstA* | phosphate-specific transport system subunit PstA | K02038 | COG0581 |
| *pstB* | phosphate-specific transport system subunit PstB | K02036 | COG1117 |
| *pstC* | phosphate-specific transport system subunit PstC | K02037 | COG0573 |
| *pstS* | phosphate-specific transport system subunit PstS | K02040 | COG0226 |
| *ugpA* | glycerol-3-phosphate transporter subunit UgpA | K05814 | COG1175 |
| *ugpB* | glycerol-3-phosphate transporter subunit UgpB | K05813 | COG1653 |
| *ugpC* | glycerol-3-phosphate transporter subunit UgpC | K05816 | COG3839 |
| *ugpE* | glycerol-3-phosphate transporter subunit UgpE | K05815 | COG0395 |
| *pit* | phosphate inorganic transporter | K03306 | COG0306 |
| P regulation | | | |
| *phoB* | phosphate regulon response regulator (PhoB) | K07657 | COG0745 |
| *phoR* | phosphate regulon sensor histidine kinase (PhoR) | K07636 | COG0642 |
| *phoU* | PhoR/PhoB inhibitor protein | K02039 | COG0704 |

**Table S3.** Pearson correlations between the concentrations of bioavailable soil P and the relative abundances of the P-related genes selected by Random Forest analysis.

| **Gene** | ***r*** | ***P* value** |
| --- | --- | --- |
| *gcd* | 0.83 | 0.0000 |
| *phnW* | 0.84 | 0.0000 |
| *phoA* | 0.83 | 0.0000 |
| *ppx* | 0.60 | 0.0085 |
| *phoX* | 0.84 | 0.0000 |
| *phoN* | 0.77 | 0.0002 |
| *olpA* | 0.81 | 0.0000 |
| *phoD* | 0.79 | 0.0001 |
| *phnN* | 0.56 | 0.0151 |
| *phnJ* | 0.62 | 0.0056 |
| *phnG* | 0.48 | 0.0444 |
| *phnM* | 0.54 | 0.0216 |
| *phnP* | 0.55 | 0.0176 |
| *phnX* | 0.44 | 0.0675 |
| *phnH* | 0.51 | 0.0314 |

**Table S4.** Overview of the 424 high-quality genome bins reconstructed in this study.

| **Bins** | **Taxonomy** | **No. of scaffolds** | **No. of genes** | **size (M)** | **GC (%)** | **Completeness (%)** | **Contamination (%)** | **KEGG (%)** | **COG (%)** |
| --- | --- | --- | --- | --- | --- | --- | --- | --- | --- |
| ALRT3_3.63 | Bacteria | 300 | 6098 | 7.10 | 67.77 | 90.49 | 1.71 | 34.72 | 72.63 |
| ALRT4_1.19 | Bacteria | 519 | 6042 | 6.72 | 70.68 | 96.47 | 2.74 | 36.69 | 77.46 |
| ULRT4_1.29 | Bacteria | 48 | 3096 | 3.63 | 56.85 | 98.10 | 0.00 | 46.48 | 81.46 |
| ULRT4_1.77 | Bacteria | 239 | 4196 | 5.11 | 59.67 | 97.25 | 1.72 | 42.47 | 80.22 |
| ULRT4_2.107 | Bacteria | 170 | 4008 | 4.48 | 59.25 | 90.94 | 0.00 | 41.97 | 74.53 |
| ULRT4_2.48 | Bacteria | 465 | 5139 | 5.82 | 62.65 | 93.53 | 0.00 | 38.35 | 77.08 |
| ULRT4_2.74 | Bacteria | 72 | 4196 | 5.00 | 56.55 | 99.73 | 0.00 | 40.35 | 77.57 |
| ULRT4_3.13 | Bacteria | 36 | 3733 | 4.49 | 58.54 | 98.06 | 4.31 | 43.34 | 79.91 |
| ULRT4_3.50 | Bacteria | 48 | 6780 | 8.08 | 63.34 | 93.91 | 2.61 | 33.79 | 78.17 |
| ULRT4_3.75 | Bacteria | 25 | 3947 | 4.75 | 61.70 | 98.06 | 0.86 | 40.92 | 77.35 |
| ULRT3_1.133 | Bacteria | 71 | 3334 | 3.87 | 60.46 | 97.65 | 0.85 | 44.00 | 78.88 |
| ULRT3_3.130 | Bacteria | 68 | 3358 | 4.04 | 62.86 | 99.15 | 0.85 | 45.74 | 80.88 |
| ALRT3_1.33 | Bacteria | 408 | 6048 | 6.12 | 63.51 | 94.65 | 1.16 | 44.48 | 80.94 |
| ALRT3_3.36 | Bacteria | 67 | 3379 | 3.54 | 68.53 | 93.27 | 0.88 | 45.22 | 78.22 |
| ALRT4_1.43 | Bacteria | 191 | 2615 | 2.45 | 66.09 | 96.22 | 0.74 | 51.20 | 80.99 |
| ULRT4_1.51 | Bacteria | 222 | 3007 | 3.03 | 67.47 | 95.58 | 0.25 | 57.70 | 86.46 |
| ULRT3_1.83 | Bacteria | 268 | 4096 | 3.85 | 65.74 | 94.76 | 0.66 | 42.82 | 74.63 |
| ULRT3_2.126 | Bacteria | 36 | 3015 | 3.17 | 66.52 | 94.90 | 0.50 | 57.71 | 87.69 |
| ULRT3_3.52 | Bacteria | 157 | 6058 | 6.53 | 65.78 | 97.51 | 1.24 | 42.70 | 79.80 |
| ALRT4_2.11 | Bacteria | 468 | 4463 | 5.31 | 50.99 | 90.10 | 0.06 | 32.04 | 74.70 |
| ULRT3_1.11 | Bacteria | 43 | 5550 | 6.56 | 43.16 | 98.94 | 0.00 | 32.27 | 73.98 |
| ULRT3_1.160 | Bacteria | 64 | 4621 | 5.43 | 52.34 | 98.51 | 1.34 | 35.25 | 77.65 |
| ULRT3_1.60 | Bacteria | 252 | 5503 | 6.26 | 39.87 | 98.52 | 0.21 | 34.87 | 74.32 |
| ULRT3_1.79 | Bacteria | 21 | 5256 | 6.28 | 44.66 | 99.51 | 0.49 | 33.92 | 75.84 |
| ALRT3_2.16 | Bacteria | 94 | 5063 | 5.61 | 66.24 | 99.62 | 1.57 | 50.03 | 85.88 |
| ALRT4_1.10 | Bacteria | 333 | 6305 | 7.37 | 64.38 | 93.51 | 1.47 | 31.40 | 71.25 |
| ALRT3_1.7 | Bacteria | 117 | 3885 | 4.17 | 66.56 | 96.82 | 1.05 | 49.01 | 82.52 |
| ALRT3_3.35 | Bacteria | 204 | 3841 | 4.07 | 64.34 | 92.16 | 0.27 | 49.26 | 82.45 |
| ALRT3_3.62 | Bacteria | 178 | 4436 | 4.95 | 62.43 | 96.51 | 2.20 | 49.46 | 82.89 |
| ALRT3_3.83 | Bacteria | 205 | 2880 | 2.98 | 66.12 | 93.82 | 1.15 | 52.01 | 82.74 |
| ALRT4_1.24 | Bacteria | 325 | 4440 | 4.62 | 65.26 | 93.25 | 1.92 | 45.52 | 79.44 |
| ALRT4_2.13 | Bacteria | 331 | 4666 | 4.78 | 65.58 | 93.90 | 2.97 | 41.30 | 75.63 |
| ALRT4_2.36 | Bacteria | 251 | 4217 | 4.47 | 66.13 | 93.27 | 2.63 | 45.96 | 80.37 |
| ALRT4_3.1 | Bacteria | 511 | 5111 | 5.31 | 65.17 | 96.11 | 3.39 | 44.39 | 82.55 |
| ULRT4_3.172 | Bacteria | 6 | 2574 | 2.80 | 58.64 | 96.47 | 2.84 | 57.42 | 83.53 |
| ULRT3_2.14 | Bacteria | 140 | 3598 | 3.86 | 67.69 | 97.33 | 0.74 | 51.47 | 81.66 |
| ULRT3_2.161 | Bacteria | 38 | 2935 | 3.26 | 69.67 | 95.09 | 2.20 | 46.37 | 78.23 |
| ULRT3_2.4 | Bacteria | 349 | 3598 | 3.80 | 71.66 | 90.11 | 0.20 | 41.38 | 75.96 |
| UT4_2.26 | Bacteria | 204 | 4801 | 6.30 | 57.49 | 97.92 | 1.16 | 32.66 | 70.11 |
| UT4_1.90 | Bacteria | 49 | 2132 | 2.43 | 66.99 | 100.00 | 1.28 | 49.95 | 82.60 |
| UT4_2.120 | Bacteria | 53 | 2123 | 2.41 | 66.96 | 100.00 | 1.28 | 49.93 | 82.71 |
| UT4_3.155 | Bacteria | 47 | 2140 | 2.44 | 66.95 | 100.00 | 1.28 | 49.58 | 82.38 |
| ULRT4_1.153 | Bacteria | 29 | 2136 | 2.45 | 66.93 | 100.00 | 0.43 | 49.53 | 82.44 |
| UT3_2.27 | Bacteria | 193 | 3474 | 3.74 | 67.06 | 100.00 | 0.43 | 41.31 | 75.13 |
| ULRT4_3.19 | Bacteria | 75 | 2935 | 3.38 | 56.77 | 100.00 | 1.10 | 41.50 | 72.30 |
| ULRT4_2.162 | Bacteria | 25 | 4078 | 4.94 | 43.09 | 100.00 | 2.69 | 41.42 | 81.51 |
| ULRT3_2.115 | Bacteria | 83 | 3194 | 3.84 | 54.79 | 99.78 | 0.00 | 50.78 | 83.81 |
| ULRT3_2.66 | Bacteria | 9 | 4522 | 5.22 | 40.23 | 99.75 | 0.25 | 35.47 | 75.98 |
| ULRT4_3.100 | Bacteria | 58 | 4333 | 5.14 | 56.64 | 99.73 | 0.86 | 39.90 | 76.99 |
| ULRT3_2.82 | Bacteria | 13 | 2621 | 2.71 | 67.49 | 99.59 | 1.10 | 50.86 | 81.84 |
| UT3_1.46 | Bacteria | 34 | 2113 | 2.42 | 67.04 | 99.57 | 0.43 | 49.22 | 82.16 |
| ULRT4_2.81 | Archaea | 4 | 2096 | 1.98 | 45.02 | 99.55 | 0.81 | 42.99 | 73.62 |
| ULRT4_2.87 | Bacteria | 6 | 2671 | 2.81 | 60.19 | 99.52 | 0.79 | 58.14 | 86.11 |
| ULRT4_2.101 | Bacteria | 23 | 3100 | 3.75 | 39.72 | 99.42 | 0.38 | 38.45 | 75.29 |
| ALRT3_3.17 | Bacteria | 39 | 4040 | 4.18 | 64.95 | 99.39 | 0.68 | 50.97 | 85.15 |
| ULRT4_3.83 | Bacteria | 83 | 4318 | 5.68 | 65.81 | 99.32 | 2.74 | 40.55 | 77.35 |
| ULRT3_3.8 | Bacteria | 66 | 3259 | 3.66 | 67.64 | 99.20 | 0.34 | 56.83 | 86.99 |
| ALRT3_3.65 | Bacteria | 16 | 2963 | 3.16 | 70.49 | 99.20 | 0.46 | 50.49 | 83.77 |
| ULRT4_3.8 | Bacteria | 19 | 2209 | 2.28 | 66.49 | 99.15 | 1.28 | 51.92 | 82.84 |
| ULRT3_2.83 | Bacteria | 26 | 2246 | 2.30 | 66.36 | 99.15 | 1.28 | 52.14 | 82.41 |
| UT4_1.117 | Bacteria | 105 | 2539 | 3.07 | 72.04 | 99.15 | 1.71 | 48.84 | 77.35 |
| UT3_3.40 | Bacteria | 171 | 4461 | 4.59 | 70.61 | 99.15 | 0.85 | 35.17 | 73.84 |
| ULRT4_2.8 | Bacteria | 21 | 3933 | 4.06 | 63.22 | 99.13 | 1.74 | 50.42 | 83.98 |
| ULRT3_3.50 | Bacteria | 53 | 2406 | 2.55 | 56.24 | 99.00 | 0.25 | 53.53 | 78.10 |
| ULRT4_2.120 | Bacteria | 25 | 2921 | 3.16 | 66.07 | 99.00 | 0.25 | 57.55 | 87.20 |
| ULRT3_1.7 | Bacteria | 34 | 2902 | 2.89 | 69.24 | 98.99 | 0.63 | 47.28 | 81.67 |
| ULRT4_2.84 | Bacteria | 15 | 3151 | 3.84 | 43.04 | 98.87 | 0.19 | 41.29 | 78.36 |
| ULRT3_1.58 | Bacteria | 138 | 3574 | 4.52 | 54.63 | 98.86 | 0.00 | 38.25 | 67.91 |
| ULRT3_3.101 | Bacteria | 73 | 3543 | 4.53 | 54.62 | 98.86 | 0.00 | 38.58 | 67.94 |
| ULRT3_2.48 | Bacteria | 55 | 3552 | 4.58 | 54.62 | 98.86 | 0.00 | 38.49 | 68.16 |
| ULRT4_2.41 | Bacteria | 6 | 2659 | 2.70 | 36.48 | 98.85 | 0.10 | 47.20 | 75.25 |
| ULRT3_1.161 | Bacteria | 127 | 2931 | 3.29 | 56.87 | 98.84 | 0.10 | 42.92 | 72.81 |
| ULRT4_2.32 | Bacteria | 17 | 2530 | 2.68 | 60.93 | 98.81 | 0.63 | 61.30 | 86.92 |
| ULRT4_3.51 | Archaea | 84 | 1830 | 1.69 | 45.25 | 98.75 | 0.00 | 47.21 | 78.91 |
| ULRT4_2.72 | Bacteria | 60 | 2989 | 3.68 | 43.86 | 98.68 | 0.38 | 42.22 | 79.99 |
| UT4_2.99 | Bacteria | 239 | 3093 | 3.29 | 73.23 | 98.67 | 2.56 | 44.33 | 80.25 |
| ULRT4_2.196 | Bacteria | 168 | 3521 | 4.44 | 54.64 | 98.66 | 0.00 | 38.99 | 68.42 |
| UT3_3.55 | Bacteria | 122 | 2749 | 3.05 | 71.06 | 98.64 | 0.00 | 44.13 | 80.14 |
| ULRT4_1.61 | Bacteria | 135 | 2072 | 2.16 | 48.35 | 98.62 | 0.43 | 52.65 | 82.53 |
| ULRT4_1.156 | Bacteria | 32 | 1754 | 1.79 | 39.90 | 98.56 | 0.57 | 55.13 | 73.95 |
| ULRT4_3.64 | Bacteria | 52 | 5305 | 6.18 | 39.94 | 98.52 | 0.49 | 34.76 | 74.50 |
| ULRT4_1.15 | Bacteria | 62 | 2511 | 2.62 | 56.28 | 98.51 | 0.00 | 53.13 | 76.86 |
| ULRT4_2.106 | Bacteria | 294 | 6539 | 8.23 | 62.54 | 98.47 | 0.57 | 30.92 | 69.61 |
| ULRT4_2.138 | Bacteria | 94 | 4460 | 5.16 | 64.42 | 98.35 | 0.65 | 44.24 | 81.19 |
| ALRT3_3.95 | Bacteria | 23 | 2992 | 3.21 | 68.42 | 98.33 | 0.62 | 50.47 | 82.19 |
| ULRT3_2.139 | Bacteria | 90 | 3148 | 3.75 | 62.93 | 98.29 | 1.28 | 46.41 | 81.19 |
| UT3_2.47 | Bacteria | 31 | 2939 | 3.23 | 60.07 | 98.29 | 2.99 | 46.07 | 79.38 |
| UT3_2.2 | Bacteria | 94 | 1984 | 2.15 | 48.42 | 98.29 | 0.43 | 54.33 | 84.73 |
| UT3_3.77 | Bacteria | 23 | 2858 | 3.15 | 60.06 | 98.29 | 2.99 | 47.24 | 80.27 |
| UT4_1.32 | Bacteria | 154 | 3709 | 3.89 | 67.07 | 98.29 | 2.42 | 40.93 | 77.06 |
| ULRT4_1.54 | Bacteria | 220 | 4189 | 4.49 | 65.67 | 98.29 | 2.14 | 41.01 | 76.58 |
| ULRT3_2.174 | Bacteria | 148 | 3490 | 3.86 | 75.14 | 98.29 | 0.43 | 41.83 | 78.42 |
| ULRT3_3.88 | Bacteria | 30 | 2976 | 3.30 | 71.19 | 98.29 | 2.14 | 45.97 | 82.59 |
| ULRT4_1.134 | Bacteria | 109 | 3827 | 4.20 | 72.45 | 98.29 | 0.43 | 43.06 | 80.11 |
| ULRT4_2.189 | Bacteria | 74 | 3903 | 4.08 | 66.28 | 98.29 | 0.00 | 51.47 | 85.47 |
| ULRT3_2.92 | Bacteria | 137 | 2749 | 2.92 | 66.61 | 98.26 | 0.25 | 59.26 | 88.07 |
| UT3_2.52 | Bacteria | 95 | 2851 | 3.37 | 60.98 | 98.22 | 0.00 | 47.95 | 82.08 |
| UT3_2.69 | Bacteria | 141 | 2711 | 2.97 | 70.94 | 98.21 | 0.28 | 45.59 | 81.34 |
| UT4_1.130 | Bacteria | 239 | 2681 | 2.73 | 69.94 | 98.20 | 2.14 | 45.32 | 83.70 |
| ULRT4_2.135 | Bacteria | 62 | 2376 | 2.41 | 61.43 | 98.17 | 1.22 | 57.24 | 82.32 |
| ULRT3_2.99 | Bacteria | 6 | 2672 | 2.79 | 60.18 | 98.17 | 0.00 | 57.11 | 83.05 |
| ULRT3_2.54 | Bacteria | 40 | 2835 | 2.77 | 57.26 | 98.10 | 0.00 | 54.99 | 80.53 |
| UT3_3.39 | Archaea | 25 | 1509 | 1.49 | 43.73 | 98.08 | 0.81 | 53.15 | 84.49 |
| ULRT3_1.116 | Bacteria | 257 | 6661 | 8.15 | 45.47 | 98.03 | 0.12 | 31.78 | 74.84 |
| UT4_2.89 | Bacteria | 84 | 2384 | 2.50 | 56.18 | 98.01 | 0.00 | 54.53 | 78.06 |
| ULRT4_2.195 | Bacteria | 88 | 5811 | 6.33 | 65.32 | 97.96 | 0.67 | 50.39 | 84.12 |
| ULRT4_2.198 | Bacteria | 195 | 3544 | 4.28 | 65.49 | 97.95 | 0.68 | 47.55 | 82.70 |
| ULRT3_1.80 | Archaea | 40 | 2001 | 1.97 | 44.20 | 97.94 | 0.00 | 44.48 | 73.61 |
| ULRT3_2.127 | Bacteria | 119 | 3928 | 4.04 | 63.36 | 97.93 | 0.65 | 51.86 | 84.88 |
| ULRT3_1.21 | Bacteria | 156 | 7092 | 7.93 | 71.19 | 97.86 | 1.07 | 35.48 | 77.78 |
| UT4_2.79 | Bacteria | 281 | 2512 | 2.94 | 72.03 | 97.86 | 2.14 | 49.04 | 77.55 |
| UT3_3.83 | Archaea | 38 | 1521 | 1.49 | 46.55 | 97.85 | 0.00 | 53.98 | 86.59 |
| ULRT4_2.63 | Bacteria | 28 | 1289 | 1.34 | 34.91 | 97.85 | 0.00 | 52.52 | 68.81 |
| ULRT4_2.155 | Bacteria | 18 | 3545 | 3.78 | 61.28 | 97.84 | 0.00 | 64.68 | 87.11 |
| ALRT3_1.2 | Bacteria | 151 | 6620 | 6.80 | 71.69 | 97.75 | 0.53 | 35.30 | 79.41 |
| ULRT3_2.51 | Bacteria | 122 | 3054 | 3.26 | 67.13 | 97.64 | 0.75 | 57.43 | 86.90 |
| ULRT4_3.152 | Bacteria | 87 | 3426 | 3.40 | 62.57 | 97.61 | 0.88 | 53.53 | 85.11 |
| ULRT4_2.46 | Bacteria | 33 | 3787 | 4.19 | 57.19 | 97.60 | 0.00 | 48.01 | 81.59 |
| UT3_1.14 | Bacteria | 72 | 2571 | 2.70 | 64.11 | 97.56 | 1.22 | 58.77 | 82.77 |
| ULRT3_3.106 | Bacteria | 54 | 2445 | 2.45 | 61.41 | 97.56 | 1.22 | 55.91 | 80.94 |
| ULRT4_2.165 | Bacteria | 112 | 3682 | 4.55 | 42.62 | 97.52 | 0.50 | 32.81 | 74.58 |
| UT4_3.49 | Archaea | 61 | 1806 | 1.71 | 37.17 | 97.52 | 2.44 | 48.01 | 79.96 |
| UT3_1.39 | Bacteria | 113 | 2518 | 2.68 | 58.57 | 97.44 | 1.28 | 45.99 | 77.52 |
| UT3_3.29 | Bacteria | 123 | 1970 | 2.10 | 48.39 | 97.44 | 0.43 | 54.52 | 83.91 |
| ULRT4_2.27 | Bacteria | 136 | 3298 | 3.44 | 74.49 | 97.44 | 0.43 | 40.15 | 78.87 |
| UT3_1.85 | Bacteria | 82 | 2845 | 3.33 | 73.25 | 97.44 | 0.43 | 45.94 | 79.61 |
| UT4_2.23 | Bacteria | 274 | 2909 | 3.13 | 67.30 | 97.44 | 1.28 | 44.52 | 76.04 |
| UT3_1.15 | Bacteria | 110 | 3644 | 3.90 | 70.88 | 97.44 | 1.71 | 38.06 | 76.40 |
| UT3_2.15 | Bacteria | 82 | 3089 | 3.32 | 71.09 | 97.44 | 0.85 | 40.56 | 78.15 |
| UT3_3.14 | Bacteria | 148 | 2440 | 2.58 | 58.59 | 97.44 | 0.43 | 46.97 | 77.83 |
| ULRT3_2.44 | Bacteria | 85 | 2284 | 2.40 | 46.02 | 97.44 | 0.43 | 50.53 | 81.22 |
| ULRT3_3.46 | Bacteria | 189 | 4675 | 4.96 | 69.05 | 97.44 | 0.27 | 38.37 | 79.74 |
| ULRT4_1.31 | Bacteria | 94 | 3090 | 3.27 | 74.81 | 97.44 | 0.43 | 41.68 | 79.48 |
| UT3_3.35 | Bacteria | 90 | 2992 | 3.48 | 73.22 | 97.44 | 0.43 | 45.22 | 79.51 |
| UT3_3.57 | Bacteria | 153 | 3002 | 3.26 | 72.45 | 97.44 | 0.00 | 45.14 | 79.68 |
| ULRT3_3.127 | Bacteria | 98 | 2744 | 2.90 | 66.56 | 97.42 | 0.00 | 61.52 | 89.61 |
| ALRT3_3.53 | Bacteria | 8 | 2289 | 2.35 | 68.92 | 97.41 | 1.01 | 44.39 | 79.69 |
| ULRT3_1.66 | Bacteria | 64 | 3160 | 3.04 | 68.01 | 97.39 | 0.14 | 45.66 | 77.15 |
| ALRT3_3.44 | Bacteria | 167 | 5345 | 6.53 | 60.78 | 97.38 | 2.25 | 31.60 | 67.67 |
| ULRT4_3.170 | Bacteria | 12 | 3095 | 3.41 | 68.36 | 97.37 | 0.00 | 49.01 | 79.35 |
| ULRT4_3.174 | Bacteria | 111 | 4406 | 4.77 | 69.92 | 97.37 | 2.63 | 44.76 | 77.39 |
| ULRT4_2.83 | Bacteria | 16 | 1955 | 2.34 | 45.17 | 97.28 | 0.68 | 48.39 | 74.48 |
| ULRT4_2.192 | Bacteria | 136 | 4248 | 5.11 | 57.52 | 97.26 | 1.37 | 40.98 | 76.48 |
| ULRT4_2.190 | Bacteria | 112 | 2035 | 2.06 | 36.49 | 97.18 | 0.56 | 46.73 | 75.14 |
| UT3_1.3 | Bacteria | 149 | 2438 | 2.68 | 71.18 | 97.14 | 1.71 | 47.42 | 81.58 |
| UT3_1.22 | Bacteria | 144 | 2969 | 3.27 | 67.20 | 97.08 | 0.43 | 43.99 | 76.32 |
| UT3_2.7 | Archaea | 54 | 1515 | 1.46 | 46.27 | 97.04 | 0.00 | 53.00 | 85.48 |
| ALRT4_2.17 | Bacteria | 39 | 3743 | 4.75 | 42.70 | 97.03 | 0.00 | 32.09 | 73.87 |
| UT3_2.43 | Bacteria | 291 | 3827 | 4.09 | 71.91 | 97.01 | 0.00 | 40.03 | 75.49 |
| ULRT3_1.50 | Bacteria | 18 | 2448 | 2.42 | 57.47 | 97.00 | 0.71 | 59.60 | 84.72 |
| UT3_3.12 | Archaea | 31 | 1823 | 1.82 | 44.22 | 97.00 | 0.81 | 46.52 | 75.86 |
| ALRT3_3.48 | Bacteria | 79 | 2510 | 2.51 | 69.59 | 96.98 | 0.72 | 41.12 | 76.89 |
| ALRT4_3.37 | Bacteria | 8 | 2548 | 2.59 | 69.11 | 96.98 | 1.72 | 41.09 | 77.59 |
| ULRT4_1.129 | Bacteria | 146 | 2565 | 2.72 | 67.25 | 96.97 | 1.97 | 60.04 | 86.90 |
| ALRT4_3.30 | Bacteria | 387 | 6690 | 6.69 | 71.74 | 96.96 | 0.90 | 35.74 | 79.60 |
| ULRT4_2.110 | Bacteria | 146 | 2911 | 3.55 | 60.22 | 96.96 | 0.00 | 47.20 | 83.00 |
| UT4_2.80 | Bacteria | 78 | 2367 | 2.34 | 61.46 | 96.95 | 1.22 | 56.49 | 80.86 |
| ULRT4_2.144 | Bacteria | 125 | 2877 | 2.95 | 63.78 | 96.95 | 0.61 | 54.88 | 78.73 |
| ULRT3_2.175 | Bacteria | 506 | 8783 | 9.07 | 70.16 | 96.95 | 0.44 | 31.60 | 75.65 |
| ULRT4_1.82 | Bacteria | 188 | 2815 | 3.22 | 73.20 | 96.92 | 0.43 | 46.32 | 80.04 |
| ULRT4_3.80 | Bacteria | 81 | 3278 | 3.46 | 62.11 | 96.89 | 1.00 | 52.96 | 84.75 |
| UT3_3.10 | Bacteria | 187 | 4775 | 6.31 | 57.47 | 96.86 | 2.43 | 32.61 | 69.91 |
| ULRT3_2.85 | Bacteria | 38 | 2492 | 2.74 | 69.44 | 96.86 | 0.86 | 61.56 | 87.36 |
| ULRT3_2.130 | Bacteria | 87 | 2682 | 2.87 | 66.51 | 96.78 | 0.00 | 62.79 | 90.04 |
| ULRT4_1.74 | Bacteria | 238 | 3754 | 3.74 | 71.19 | 96.77 | 0.00 | 50.80 | 85.67 |
| UT4_3.91 | Bacteria | 78 | 2275 | 2.30 | 61.51 | 96.75 | 1.22 | 58.02 | 82.24 |
| UT3_3.42 | Bacteria | 41 | 3073 | 3.45 | 66.45 | 96.74 | 3.29 | 56.56 | 83.21 |
| ULRT4_1.161 | Bacteria | 50 | 2768 | 3.13 | 66.76 | 96.74 | 3.17 | 60.40 | 85.40 |
| ULRT4_3.85 | Bacteria | 23 | 4469 | 5.19 | 62.60 | 96.74 | 0.65 | 43.90 | 79.41 |
| ULRT3_3.36 | Bacteria | 785 | 7265 | 8.57 | 65.61 | 96.65 | 0.07 | 29.15 | 68.75 |
| ULRT4_2.150 | Bacteria | 61 | 3271 | 3.49 | 64.39 | 96.64 | 0.00 | 43.56 | 77.59 |
| UT4_3.88 | Bacteria | 107 | 2524 | 2.74 | 69.45 | 96.58 | 1.28 | 48.97 | 83.95 |
| UT3_2.31 | Bacteria | 63 | 2719 | 2.99 | 70.05 | 96.58 | 1.28 | 49.21 | 80.43 |
| UT3_3.41 | Bacteria | 38 | 2623 | 2.91 | 71.93 | 96.58 | 2.14 | 50.13 | 82.39 |
| UT4_3.136 | Bacteria | 166 | 3587 | 3.82 | 71.08 | 96.58 | 3.43 | 39.98 | 77.61 |
| ULRT4_3.150 | Bacteria | 30 | 2021 | 2.01 | 71.10 | 96.58 | 0.43 | 50.82 | 81.94 |
| UT3_1.6 | Bacteria | 62 | 2631 | 2.89 | 69.47 | 96.58 | 1.38 | 47.85 | 82.63 |
| ULRT4_1.67 | Bacteria | 64 | 2946 | 3.23 | 70.10 | 96.58 | 1.28 | 47.25 | 79.33 |
| UT3_2.53 | Bacteria | 238 | 3565 | 3.77 | 70.11 | 96.58 | 0.85 | 42.30 | 76.55 |
| ULRT4_3.88 | Bacteria | 135 | 3513 | 4.35 | 65.66 | 96.58 | 2.22 | 48.08 | 83.29 |
| ALRT4_1.39 | Bacteria | 20 | 2568 | 2.62 | 69.62 | 96.55 | 0.00 | 41.36 | 75.74 |
| ULRT4_2.26 | Bacteria | 204 | 2217 | 2.37 | 66.94 | 96.54 | 1.28 | 48.94 | 80.74 |
| ULRT3_3.142 | Bacteria | 101 | 2818 | 3.01 | 66.44 | 96.52 | 0.25 | 58.77 | 88.25 |
| ULRT3_1.67 | Bacteria | 340 | 7360 | 7.50 | 64.53 | 96.51 | 4.28 | 42.69 | 79.93 |
| UT4_3.96 | Bacteria | 112 | 1804 | 1.91 | 38.30 | 96.51 | 0.00 | 57.48 | 79.21 |
| ULRT4_3.108 | Bacteria | 32 | 2489 | 2.61 | 60.95 | 96.46 | 1.35 | 60.71 | 86.62 |
| UT3_2.25 | Archaea | 45 | 1586 | 1.53 | 43.87 | 96.46 | 0.81 | 49.24 | 79.63 |
| ULRT4_3.9 | Bacteria | 42 | 2322 | 2.29 | 57.51 | 96.39 | 1.03 | 60.90 | 85.53 |
| UT3_1.50 | Bacteria | 42 | 2357 | 2.42 | 64.42 | 96.34 | 2.13 | 60.29 | 83.96 |
| ULRT4_1.97 | Bacteria | 96 | 2328 | 2.29 | 60.17 | 96.34 | 0.20 | 56.92 | 81.44 |
| ULRT4_2.142 | Archaea | 72 | 1781 | 1.68 | 45.35 | 96.33 | 0.00 | 48.74 | 80.52 |
| UT3_2.65 | Archaea | 33 | 1795 | 1.78 | 39.57 | 96.33 | 0.00 | 46.91 | 76.10 |
| ULRT4_2.62 | Bacteria | 163 | 1797 | 1.96 | 44.07 | 96.31 | 0.00 | 49.97 | 82.08 |
| UT3_1.28 | Bacteria | 95 | 3134 | 3.56 | 69.25 | 96.30 | 1.28 | 44.29 | 77.03 |
| UT4_3.126 | Bacteria | 159 | 2683 | 2.95 | 67.02 | 96.30 | 4.44 | 61.57 | 85.69 |
| UT3_2.50 | Archaea | 53 | 1548 | 1.44 | 39.20 | 96.30 | 0.00 | 53.42 | 87.21 |
| UT3_2.70 | Bacteria | 133 | 2652 | 2.81 | 66.55 | 96.27 | 0.00 | 59.62 | 88.27 |
| UT3_3.19 | Bacteria | 144 | 2692 | 2.86 | 66.58 | 96.27 | 0.25 | 59.29 | 88.19 |
| ULRT3_2.64 | Bacteria | 148 | 3688 | 3.72 | 71.03 | 96.27 | 0.00 | 50.49 | 85.60 |
| ULRT4_2.6 | Bacteria | 130 | 2649 | 2.49 | 59.45 | 96.23 | 0.31 | 56.02 | 80.86 |
| UT3_2.62 | Archaea | 97 | 1672 | 1.64 | 44.57 | 96.19 | 0.00 | 50.90 | 81.22 |
| ULRT3_3.31 | Archaea | 153 | 2367 | 2.14 | 41.73 | 96.19 | 1.61 | 40.60 | 72.62 |
| ULRT3_1.85 | Bacteria | 253 | 3526 | 4.07 | 59.86 | 96.15 | 1.71 | 43.22 | 78.36 |
| ULRT3_2.104 | Bacteria | 122 | 3184 | 3.89 | 59.97 | 96.15 | 0.00 | 45.32 | 81.34 |
| UT3_1.9 | Bacteria | 83 | 2670 | 2.87 | 70.34 | 96.15 | 2.14 | 48.80 | 79.66 |
| ULRT4_1.151 | Bacteria | 30 | 3225 | 3.90 | 67.73 | 96.15 | 1.03 | 42.11 | 76.81 |
| ALRT3_3.43 | Bacteria | 18 | 2395 | 2.47 | 69.73 | 96.12 | 0.86 | 42.63 | 77.04 |
| ULRT4_3.153 | Bacteria | 70 | 3151 | 3.50 | 41.69 | 96.10 | 1.43 | 34.75 | 71.82 |
| ULRT3_2.89 | Bacteria | 398 | 7204 | 8.98 | 61.49 | 96.03 | 0.00 | 28.94 | 67.14 |
| ULRT4_1.108 | Bacteria | 125 | 3511 | 4.44 | 54.60 | 96.02 | 0.00 | 38.59 | 68.04 |
| UT3_1.80 | Bacteria | 39 | 3185 | 3.19 | 58.79 | 96.00 | 0.00 | 45.65 | 78.46 |
| UT3_3.46 | Bacteria | 45 | 3441 | 3.43 | 58.69 | 96.00 | 0.00 | 43.39 | 76.20 |
| UT3_2.58 | Bacteria | 40 | 3271 | 3.28 | 58.75 | 96.00 | 0.00 | 44.91 | 77.90 |
| ULRT4_2.121 | Bacteria | 82 | 2915 | 3.20 | 27.03 | 95.97 | 0.00 | 46.96 | 76.50 |
| UT3_3.53 | Bacteria | 135 | 3121 | 3.28 | 67.59 | 95.97 | 1.02 | 45.66 | 77.64 |
| UT3_2.5 | Archaea | 23 | 1732 | 1.72 | 38.38 | 95.93 | 0.00 | 45.55 | 76.10 |
| UT3_1.55 | Archaea | 102 | 1899 | 1.84 | 42.00 | 95.93 | 0.00 | 47.29 | 77.83 |
| UT3_1.53 | Bacteria | 152 | 2916 | 3.18 | 66.57 | 95.91 | 1.71 | 58.54 | 84.12 |
| ULRT3_1.92 | Bacteria | 257 | 3386 | 3.17 | 63.76 | 95.89 | 2.90 | 51.39 | 82.37 |
| ULRT3_2.123 | Bacteria | 148 | 3811 | 3.97 | 71.67 | 95.87 | 0.33 | 54.76 | 88.90 |
| UT3_2.4 | Bacteria | 53 | 2394 | 2.54 | 68.80 | 95.83 | 0.93 | 44.95 | 77.40 |
| ULRT4_2.109 | Bacteria | 46 | 2614 | 2.76 | 68.56 | 95.83 | 0.93 | 42.35 | 75.75 |
| ULRT4_2.66 | Bacteria | 236 | 2840 | 3.08 | 48.68 | 95.81 | 0.93 | 47.92 | 82.01 |
| ULRT4_2.114 | Archaea | 115 | 2555 | 2.38 | 37.89 | 95.79 | 0.00 | 37.61 | 66.14 |
| ULRT3_2.103 | Bacteria | 97 | 4221 | 4.80 | 67.12 | 95.74 | 4.53 | 51.91 | 83.75 |
| UT3_2.60 | Bacteria | 175 | 2368 | 2.46 | 58.73 | 95.73 | 0.71 | 46.83 | 76.31 |
| UT3_1.23 | Bacteria | 151 | 1870 | 1.96 | 48.33 | 95.73 | 0.00 | 55.94 | 84.60 |
| ULRT4_1.139 | Bacteria | 60 | 2900 | 3.03 | 63.69 | 95.73 | 1.22 | 52.59 | 79.21 |
| UT3_1.24 | Bacteria | 53 | 2034 | 2.29 | 49.38 | 95.73 | 1.28 | 46.51 | 75.61 |
| UT4_1.30 | Bacteria | 330 | 2852 | 2.97 | 73.29 | 95.73 | 2.23 | 45.69 | 80.61 |
| UT3_3.82 | Bacteria | 96 | 2710 | 2.93 | 70.25 | 95.73 | 1.28 | 48.01 | 78.93 |
| UT3_2.68 | Bacteria | 138 | 2859 | 3.13 | 72.63 | 95.73 | 0.43 | 46.00 | 79.78 |
| ULRT4_1.70 | Bacteria | 71 | 1917 | 2.15 | 49.25 | 95.73 | 0.43 | 48.83 | 77.88 |
| UT4_1.31 | Bacteria | 233 | 2590 | 2.78 | 72.47 | 95.73 | 1.57 | 51.93 | 83.32 |
| UT3_1.86 | Bacteria | 68 | 2699 | 2.82 | 68.85 | 95.71 | 0.00 | 46.61 | 78.62 |
| UT3_2.32 | Bacteria | 75 | 2776 | 2.86 | 68.96 | 95.71 | 0.00 | 45.24 | 76.59 |
| ULRT4_1.162 | Bacteria | 135 | 2815 | 2.87 | 68.84 | 95.71 | 0.00 | 45.47 | 77.83 |
| ULRT3_2.72 | Bacteria | 55 | 2973 | 3.04 | 68.81 | 95.71 | 0.00 | 43.36 | 75.14 |
| ULRT3_3.19 | Archaea | 89 | 1434 | 1.37 | 43.88 | 95.70 | 1.61 | 52.93 | 84.31 |
| ALRT4_3.38 | Bacteria | 164 | 2601 | 2.51 | 70.58 | 95.69 | 0.29 | 42.25 | 76.24 |
| ULRT4_1.73 | Bacteria | 179 | 2639 | 2.63 | 70.34 | 95.68 | 0.10 | 44.03 | 75.56 |
| UT3_1.81 | Archaea | 96 | 1983 | 1.88 | 42.15 | 95.66 | 0.00 | 44.38 | 75.04 |
| ULRT3_3.134 | Bacteria | 151 | 3063 | 3.47 | 73.14 | 95.65 | 0.85 | 45.02 | 78.84 |
| UT4_2.53 | Bacteria | 135 | 1849 | 1.80 | 63.30 | 95.65 | 0.93 | 67.66 | 87.78 |
| UT3_1.75 | Archaea | 37 | 1719 | 1.70 | 39.62 | 95.52 | 0.00 | 48.87 | 77.20 |
| ALRT4_1.22 | Bacteria | 27 | 2678 | 2.79 | 69.12 | 95.52 | 0.86 | 40.40 | 76.66 |
| UT3_3.26 | Bacteria | 115 | 2789 | 3.08 | 67.19 | 95.47 | 1.09 | 58.59 | 84.30 |
| ULRT3_2.26 | Bacteria | 396 | 5185 | 5.82 | 68.60 | 95.45 | 0.18 | 37.45 | 77.01 |
| ULRT3_3.72 | Bacteria | 232 | 3609 | 3.64 | 71.16 | 95.43 | 0.00 | 51.18 | 85.62 |
| ULRT3_1.77 | Bacteria | 51 | 3611 | 3.58 | 65.20 | 95.42 | 1.23 | 52.78 | 83.44 |
| ULRT3_2.125 | Bacteria | 63 | 1917 | 2.05 | 69.24 | 95.32 | 0.60 | 66.51 | 90.82 |
| UT3_1.68 | Bacteria | 143 | 2860 | 3.13 | 72.73 | 95.30 | 0.43 | 45.98 | 80.00 |
| ULRT3_2.60 | Bacteria | 356 | 3850 | 3.84 | 72.26 | 95.30 | 1.57 | 43.53 | 76.70 |
| ULRT3_1.75 | Bacteria | 99 | 3243 | 3.87 | 68.17 | 95.30 | 0.17 | 42.55 | 76.81 |
| UT3_2.24 | Bacteria | 55 | 2707 | 3.02 | 65.55 | 95.29 | 2.35 | 59.14 | 84.74 |
| ULRT3_2.70 | Bacteria | 312 | 7257 | 7.81 | 71.28 | 95.28 | 0.00 | 33.99 | 74.49 |
| UT3_1.71 | Bacteria | 139 | 2654 | 2.81 | 66.60 | 95.27 | 0.00 | 60.10 | 88.66 |
| UT4_1.107 | Bacteria | 130 | 2388 | 2.50 | 56.27 | 95.22 | 0.05 | 54.86 | 78.73 |
| UT3_2.26 | Bacteria | 551 | 6429 | 6.45 | 55.48 | 95.21 | 0.99 | 32.80 | 64.35 |
| ULRT3_2.17 | Bacteria | 128 | 2094 | 2.22 | 66.81 | 95.19 | 1.87 | 70.87 | 93.51 |
| UT4_1.102 | Archaea | 91 | 1419 | 1.36 | 44.68 | 95.16 | 0.06 | 51.80 | 79.92 |
| UT4_2.49 | Bacteria | 213 | 3403 | 3.52 | 64.44 | 95.00 | 2.33 | 42.87 | 74.67 |
| UT4_2.134 | Bacteria | 184 | 3022 | 3.17 | 70.18 | 94.97 | 1.28 | 46.86 | 80.64 |
| ULRT4_1.23 | Bacteria | 190 | 3138 | 3.24 | 63.41 | 94.96 | 0.05 | 44.61 | 78.33 |
| UT3_2.71 | Bacteria | 176 | 3399 | 4.23 | 61.35 | 94.88 | 0.00 | 44.75 | 78.79 |
| UT3_1.65 | Bacteria | 133 | 2341 | 2.31 | 61.50 | 94.88 | 0.00 | 56.94 | 81.12 |
| UT4_1.108 | Bacteria | 126 | 2675 | 2.96 | 59.91 | 94.87 | 2.14 | 48.93 | 81.61 |
| UT4_2.50 | Bacteria | 114 | 2806 | 3.08 | 59.94 | 94.87 | 2.99 | 47.65 | 80.76 |
| UT4_3.52 | Bacteria | 159 | 2789 | 3.03 | 59.95 | 94.87 | 2.14 | 47.54 | 81.00 |
| UT4_2.17 | Bacteria | 167 | 3128 | 3.25 | 67.20 | 94.87 | 3.28 | 42.52 | 77.81 |
| UT4_1.95 | Bacteria | 265 | 2641 | 2.73 | 65.56 | 94.87 | 4.87 | 47.67 | 77.77 |
| UT4_3.20 | Bacteria | 390 | 2920 | 3.14 | 73.15 | 94.87 | 2.31 | 44.69 | 79.21 |
| ULRT3_1.167 | Bacteria | 174 | 2221 | 2.08 | 72.71 | 94.87 | 0.57 | 50.79 | 81.67 |
| ULRT3_2.137 | Bacteria | 139 | 3763 | 4.47 | 58.52 | 94.87 | 0.00 | 43.16 | 80.92 |
| ALRT3_3.33 | Bacteria | 373 | 4422 | 4.40 | 68.87 | 94.85 | 1.04 | 48.03 | 83.18 |
| ULRT4_1.141 | Bacteria | 71 | 2300 | 2.43 | 39.45 | 94.73 | 0.58 | 51.57 | 72.26 |
| ULRT4_2.152 | Bacteria | 328 | 3850 | 3.85 | 70.38 | 94.73 | 2.87 | 56.99 | 86.86 |
| UT3_3.22 | Bacteria | 126 | 2850 | 3.23 | 69.43 | 94.72 | 0.43 | 45.44 | 76.81 |
| UT3_2.61 | Archaea | 96 | 1742 | 1.66 | 42.59 | 94.72 | 0.81 | 49.20 | 80.83 |
| ULRT3_2.65 | Archaea | 257 | 2298 | 2.01 | 43.02 | 94.68 | 0.81 | 38.86 | 69.10 |
| UT4_2.145 | Bacteria | 352 | 3443 | 3.39 | 67.65 | 94.65 | 1.32 | 43.83 | 74.24 |
| ULRT4_3.101 | Bacteria | 59 | 3325 | 3.68 | 63.97 | 94.64 | 0.79 | 46.98 | 79.49 |
| ULRT4_1.68 | Bacteria | 54 | 2380 | 2.43 | 64.40 | 94.51 | 2.13 | 58.82 | 83.03 |
| ULRT4_1.64 | Bacteria | 84 | 2579 | 3.09 | 60.46 | 94.44 | 0.85 | 47.73 | 82.51 |
| ULRT4_2.52 | Bacteria | 459 | 4932 | 5.04 | 74.79 | 94.44 | 1.71 | 40.00 | 72.65 |
| UT3_2.17 | Bacteria | 211 | 3210 | 3.58 | 69.03 | 94.35 | 1.71 | 42.96 | 75.17 |
| ULRT4_1.164 | Archaea | 109 | 2006 | 1.94 | 39.77 | 94.31 | 0.81 | 45.31 | 74.93 |
| UT3_3.37 | Archaea | 65 | 1709 | 1.70 | 37.60 | 94.18 | 0.00 | 46.46 | 75.72 |
| ULRT4_2.202 | Bacteria | 128 | 2023 | 2.09 | 32.97 | 94.18 | 0.00 | 51.16 | 78.00 |
| ULRT3_3.23 | Bacteria | 207 | 3244 | 3.60 | 69.25 | 94.16 | 1.28 | 43.96 | 75.83 |
| ULRT4_2.44 | Bacteria | 72 | 3077 | 3.41 | 36.74 | 94.13 | 1.74 | 46.86 | 79.01 |
| ULRT3_2.170 | Bacteria | 93 | 4265 | 5.12 | 66.73 | 94.06 | 1.71 | 41.38 | 79.30 |
| ULRT4_2.167 | Bacteria | 403 | 3081 | 3.62 | 55.34 | 94.05 | 0.00 | 40.93 | 68.42 |
| UT3_3.88 | Bacteria | 119 | 1754 | 1.93 | 49.10 | 94.02 | 0.43 | 50.86 | 78.45 |
| UT4_1.67 | Bacteria | 156 | 2489 | 2.67 | 69.53 | 94.02 | 1.28 | 48.94 | 84.49 |
| UT4_2.59 | Bacteria | 237 | 2520 | 2.63 | 65.61 | 94.02 | 2.14 | 47.98 | 77.90 |
| UT4_3.5 | Bacteria | 94 | 3086 | 3.20 | 71.52 | 94.02 | 1.28 | 42.61 | 80.23 |
| ULRT3_3.135 | Bacteria | 141 | 2985 | 3.33 | 67.85 | 93.99 | 1.54 | 57.76 | 87.00 |
| ULRT3_3.39 | Archaea | 71 | 1516 | 1.45 | 46.40 | 93.95 | 1.78 | 53.69 | 84.30 |
| UT3_1.51 | Bacteria | 132 | 2512 | 2.78 | 67.53 | 93.93 | 1.07 | 62.14 | 86.03 |
| UT4_3.43 | Bacteria | 284 | 3004 | 3.01 | 63.58 | 93.91 | 2.57 | 45.24 | 78.53 |
| UT3_3.25 | Archaea | 111 | 1612 | 1.56 | 42.35 | 93.91 | 0.00 | 48.33 | 79.40 |
| ULRT4_3.166 | Bacteria | 171 | 4623 | 5.06 | 75.12 | 93.91 | 0.32 | 41.27 | 75.49 |
| UT3_3.70 | Bacteria | 56 | 2688 | 3.00 | 65.43 | 93.84 | 2.35 | 59.08 | 85.19 |
| ULRT3_2.162 | Archaea | 62 | 1491 | 1.43 | 46.49 | 93.82 | 0.00 | 51.58 | 83.77 |
| ULRT3_1.16 | Bacteria | 59 | 3944 | 4.53 | 40.48 | 93.78 | 0.25 | 37.12 | 77.08 |
| UT3_1.11 | Bacteria | 350 | 3324 | 3.89 | 61.29 | 93.74 | 0.00 | 45.52 | 78.58 |
| ULRT4_2.105 | Bacteria | 24 | 2374 | 2.47 | 59.22 | 93.73 | 0.91 | 49.66 | 73.38 |
| UT4_1.137 | Bacteria | 114 | 2482 | 2.70 | 65.87 | 93.72 | 2.20 | 62.29 | 86.06 |
| ULRT3_1.33 | Bacteria | 271 | 2979 | 3.01 | 66.06 | 93.68 | 0.51 | 57.23 | 86.37 |
| ULRT4_1.101 | Archaea | 53 | 1488 | 1.41 | 43.81 | 93.64 | 0.81 | 52.02 | 82.33 |
| UT4_3.12 | Bacteria | 141 | 2907 | 3.07 | 75.04 | 93.59 | 0.95 | 43.52 | 81.84 |
| UT4_3.71 | Bacteria | 267 | 2833 | 2.86 | 68.07 | 93.54 | 1.92 | 59.41 | 87.65 |
| UT3_3.13 | Archaea | 98 | 1418 | 1.37 | 42.47 | 93.54 | 0.00 | 52.89 | 83.50 |
| UT4_3.137 | Bacteria | 276 | 3093 | 3.73 | 61.55 | 93.51 | 0.43 | 46.49 | 79.57 |
| UT3_2.19 | Bacteria | 117 | 2727 | 3.10 | 69.56 | 93.51 | 1.28 | 46.61 | 77.48 |
| UT3_2.79 | Archaea | 112 | 1548 | 1.51 | 42.32 | 93.51 | 0.00 | 49.55 | 80.49 |
| ULRT4_3.37 | Bacteria | 51 | 3522 | 3.65 | 61.11 | 93.48 | 0.00 | 45.85 | 79.81 |
| UT3_3.15 | Bacteria | 171 | 3005 | 3.24 | 69.72 | 93.45 | 0.43 | 41.50 | 73.61 |
| ULRT3_3.57 | Bacteria | 197 | 4605 | 4.98 | 71.42 | 93.45 | 0.00 | 40.00 | 78.72 |
| UT3_1.8 | Bacteria | 180 | 3713 | 3.99 | 69.25 | 93.45 | 0.43 | 39.62 | 73.93 |
| ULRT3_3.124 | Bacteria | 14 | 2294 | 2.36 | 55.68 | 93.43 | 0.91 | 50.92 | 77.20 |
| ULRT4_3.40 | Bacteria | 201 | 2813 | 2.75 | 63.84 | 93.41 | 1.67 | 58.02 | 84.82 |
| ULRT4_1.91 | Bacteria | 175 | 3120 | 3.32 | 71.20 | 93.35 | 0.43 | 44.04 | 82.60 |
| UT3_3.4 | Bacteria | 345 | 3345 | 3.53 | 72.74 | 93.30 | 0.43 | 44.39 | 78.62 |
| UT4_1.57 | Archaea | 59 | 1595 | 1.56 | 43.40 | 93.28 | 0.81 | 49.22 | 81.63 |
| UT3_3.76 | Bacteria | 313 | 4297 | 4.35 | 55.15 | 93.23 | 0.00 | 35.54 | 64.32 |
| ULRT4_1.100 | Bacteria | 218 | 5536 | 6.34 | 58.90 | 93.18 | 2.27 | 30.71 | 65.16 |
| ULRT3_3.4 | Bacteria | 216 | 2289 | 2.31 | 47.73 | 93.16 | 0.85 | 50.42 | 79.69 |
| UT3_2.8 | Bacteria | 83 | 2325 | 2.55 | 72.28 | 93.16 | 1.28 | 52.09 | 83.40 |
| UT4_3.29 | Bacteria | 189 | 3141 | 3.31 | 65.48 | 93.12 | 4.71 | 54.86 | 81.50 |
| ULRT4_2.92 | Bacteria | 93 | 3212 | 4.20 | 52.39 | 93.10 | 0.00 | 34.68 | 66.41 |
| ULRT3_1.159 | Bacteria | 13 | 1351 | 1.50 | 46.57 | 93.07 | 2.03 | 50.78 | 71.95 |
| UT3_1.5 | Archaea | 21 | 1342 | 1.24 | 38.89 | 93.05 | 0.00 | 55.37 | 87.41 |
| ALRT4_1.37 | Bacteria | 326 | 4566 | 5.36 | 72.59 | 93.01 | 0.00 | 34.06 | 67.85 |
| ALRT3_1.24 | Bacteria | 291 | 4024 | 3.63 | 68.57 | 92.99 | 1.01 | 49.13 | 85.83 |
| UT3_3.63 | Bacteria | 272 | 3214 | 3.44 | 68.93 | 92.97 | 0.43 | 40.91 | 73.68 |
| ULRT4_1.145 | Bacteria | 123 | 1715 | 1.79 | 62.51 | 92.96 | 0.57 | 63.21 | 86.01 |
| UT4_1.65 | Bacteria | 363 | 2619 | 2.90 | 73.49 | 92.91 | 2.56 | 45.40 | 80.11 |
| ULRT3_1.94 | Bacteria | 138 | 3391 | 3.64 | 70.80 | 92.91 | 2.05 | 42.02 | 76.64 |
| ALRT4_3.7 | Bacteria | 590 | 4884 | 4.66 | 70.44 | 92.84 | 0.92 | 49.59 | 83.89 |
| UT4_2.15 | Bacteria | 144 | 2697 | 2.97 | 73.79 | 92.81 | 2.14 | 47.13 | 80.42 |
| UT3_3.49 | Bacteria | 70 | 2674 | 2.76 | 68.91 | 92.74 | 0.00 | 46.11 | 77.52 |
| UT4_3.117 | Archaea | 146 | 1571 | 1.43 | 38.72 | 92.74 | 1.08 | 52.71 | 84.79 |
| ALRT4_2.38 | Bacteria | 159 | 8873 | 10.74 | 64.91 | 92.69 | 1.47 | 29.08 | 64.44 |
| ULRT4_2.80 | Bacteria | 94 | 2314 | 2.35 | 64.55 | 92.61 | 2.13 | 59.64 | 83.06 |
| ULRT3_1.88 | Bacteria | 177 | 2897 | 3.10 | 68.26 | 92.58 | 1.41 | 57.40 | 87.06 |
| ULRT3_3.92 | Bacteria | 192 | 3583 | 3.91 | 67.53 | 92.58 | 1.81 | 53.08 | 83.48 |
| ULRT3_1.65 | Bacteria | 77 | 2848 | 2.90 | 63.47 | 92.50 | 0.95 | 56.07 | 83.99 |
| UT4_3.56 | Bacteria | 694 | 5614 | 5.50 | 69.28 | 92.47 | 3.85 | 32.88 | 70.61 |
| ULRT3_1.59 | Bacteria | 205 | 2242 | 2.43 | 66.93 | 92.39 | 0.95 | 49.02 | 81.53 |
| UT4_1.53 | Bacteria | 180 | 2766 | 2.78 | 68.91 | 92.38 | 0.33 | 45.52 | 77.33 |
| UT4_2.111 | Bacteria | 159 | 2617 | 2.81 | 69.42 | 92.31 | 2.99 | 48.72 | 84.10 |
| UT3_3.36 | Bacteria | 170 | 2758 | 3.09 | 72.62 | 92.31 | 1.28 | 47.32 | 79.41 |
| ULRT4_1.83 | Archaea | 62 | 1703 | 1.66 | 43.33 | 92.30 | 0.81 | 48.91 | 80.39 |
| ULRT3_2.79 | Bacteria | 143 | 2093 | 2.24 | 67.77 | 92.29 | 1.54 | 63.35 | 87.91 |
| ULRT4_2.30 | Bacteria | 81 | 2611 | 2.88 | 65.30 | 92.29 | 0.14 | 55.27 | 85.71 |
| ULRT4_3.106 | Bacteria | 63 | 3499 | 3.72 | 48.31 | 92.26 | 0.65 | 48.27 | 81.62 |
| UT3_3.84 | Bacteria | 18 | 2200 | 2.25 | 55.00 | 92.22 | 0.91 | 50.86 | 75.68 |
| UT3_1.69 | Archaea | 30 | 1264 | 1.22 | 46.54 | 92.20 | 0.00 | 55.70 | 86.95 |
| ALRT3_2.18 | Bacteria | 608 | 5348 | 5.42 | 66.14 | 92.17 | 2.34 | 45.34 | 80.39 |
| UT4_3.65 | Bacteria | 313 | 3065 | 3.20 | 72.67 | 92.12 | 2.56 | 44.67 | 78.63 |
| UT4_1.139 | Bacteria | 300 | 3094 | 3.05 | 67.02 | 92.08 | 2.97 | 42.05 | 72.88 |
| UT3_3.54 | Bacteria | 64 | 2319 | 2.38 | 56.34 | 92.04 | 0.91 | 51.19 | 77.15 |
| UT3_1.16 | Bacteria | 38 | 2467 | 2.76 | 65.74 | 92.03 | 1.84 | 61.05 | 85.65 |
| UT4_3.111 | Bacteria | 208 | 3315 | 3.31 | 68.66 | 92.02 | 1.16 | 40.12 | 72.07 |
| ULRT4_1.96 | Bacteria | 171 | 2167 | 2.13 | 69.87 | 91.98 | 1.28 | 53.85 | 83.66 |
| ULRT4_2.33 | Bacteria | 67 | 2535 | 3.07 | 42.06 | 91.97 | 0.19 | 43.27 | 78.07 |
| ULRT4_1.9 | Bacteria | 161 | 1889 | 1.80 | 62.92 | 91.94 | 0.91 | 65.96 | 84.17 |
| UT3_2.56 | Bacteria | 427 | 3624 | 3.39 | 71.08 | 91.87 | 0.81 | 50.63 | 84.63 |
| UT4_3.77 | Bacteria | 360 | 5534 | 6.26 | 58.93 | 91.82 | 3.41 | 31.24 | 65.09 |
| ULRT3_2.71 | Bacteria | 289 | 4617 | 4.94 | 67.81 | 91.81 | 3.68 | 52.24 | 83.02 |
| UT4_2.144 | Bacteria | 163 | 2651 | 2.82 | 65.90 | 91.79 | 2.20 | 60.77 | 84.84 |
| ULRT3_2.182 | Bacteria | 544 | 5131 | 4.98 | 70.52 | 91.77 | 0.26 | 51.04 | 84.16 |
| UT4_3.128 | Bacteria | 23 | 1144 | 1.18 | 44.32 | 91.72 | 0.00 | 55.42 | 72.81 |
| ULRT3_3.100 | Bacteria | 146 | 3562 | 3.70 | 72.68 | 91.58 | 0.99 | 44.13 | 77.99 |
| UT4_3.104 | Bacteria | 45 | 1886 | 1.92 | 51.61 | 91.46 | 0.00 | 62.25 | 83.03 |
| ULRT3_2.178 | Bacteria | 214 | 3236 | 3.73 | 68.16 | 91.45 | 1.03 | 42.37 | 76.42 |
| UT3_1.38 | Bacteria | 340 | 2767 | 3.12 | 73.46 | 91.45 | 0.52 | 44.67 | 79.07 |
| ULRT3_2.67 | Bacteria | 50 | 3331 | 3.63 | 70.48 | 91.45 | 1.28 | 45.81 | 80.64 |
| ULRT4_3.131 | Bacteria | 9 | 2584 | 2.69 | 60.53 | 91.43 | 0.48 | 55.15 | 81.46 |
| ALRT3_3.101 | Bacteria | 249 | 3629 | 3.66 | 70.84 | 91.26 | 0.00 | 52.88 | 84.65 |
| UT3_1.57 | Bacteria | 93 | 2070 | 2.20 | 69.13 | 91.20 | 0.93 | 48.36 | 79.57 |
| ULRT4_3.122 | Bacteria | 72 | 2961 | 3.11 | 63.20 | 91.20 | 0.00 | 51.17 | 84.57 |
| UT3_3.86 | Bacteria | 250 | 3528 | 3.70 | 73.83 | 91.09 | 0.00 | 37.59 | 67.66 |
| UT3_3.31 | Bacteria | 275 | 2717 | 3.11 | 73.61 | 91.03 | 0.43 | 43.95 | 77.62 |
| ULRT4_3.141 | Bacteria | 154 | 3927 | 4.42 | 59.28 | 91.02 | 2.59 | 42.88 | 75.32 |
| ULRT3_2.120 | Bacteria | 400 | 3031 | 2.83 | 69.25 | 90.97 | 0.76 | 54.37 | 84.53 |
| UT3_1.12 | Bacteria | 352 | 2671 | 2.50 | 68.65 | 90.97 | 1.03 | 46.46 | 79.45 |
| UT4_2.83 | Bacteria | 199 | 2219 | 2.20 | 64.55 | 90.95 | 2.74 | 62.10 | 83.46 |
| UT3_1.30 | Archaea | 99 | 1870 | 1.79 | 37.79 | 90.95 | 0.00 | 43.96 | 73.53 |
| UT4_3.115 | Bacteria | 18 | 1890 | 2.06 | 62.47 | 90.94 | 1.27 | 61.48 | 84.81 |
| ALRT3_3.15 | Bacteria | 207 | 2527 | 2.39 | 71.00 | 90.92 | 0.60 | 44.95 | 77.05 |
| ULRT3_3.132 | Bacteria | 183 | 2948 | 3.53 | 60.05 | 90.85 | 0.85 | 46.40 | 81.38 |
| UT4_3.145 | Bacteria | 70 | 2078 | 2.11 | 60.13 | 90.85 | 0.91 | 60.35 | 83.78 |
| UT3_3.89 | Bacteria | 421 | 3381 | 3.42 | 67.03 | 90.84 | 0.43 | 41.29 | 72.94 |
| UT3_1.48 | Archaea | 56 | 1477 | 1.42 | 37.24 | 90.80 | 0.00 | 52.00 | 85.71 |
| ALRT3_3.57 | Bacteria | 177 | 2980 | 3.06 | 68.53 | 90.78 | 0.69 | 51.98 | 83.86 |
| UT4_3.153 | Bacteria | 68 | 1517 | 1.48 | 66.97 | 90.76 | 2.07 | 58.27 | 85.83 |
| UT4_3.112 | Bacteria | 9 | 1287 | 1.41 | 38.49 | 90.74 | 0.93 | 57.73 | 75.21 |
| UT4_3.25 | Bacteria | 228 | 2657 | 2.86 | 73.09 | 90.73 | 1.34 | 46.82 | 78.55 |
| ULRT4_2.204 | Bacteria | 583 | 4553 | 5.32 | 65.68 | 90.72 | 0.00 | 38.15 | 73.75 |
| UT3_1.66 | Bacteria | 331 | 3774 | 3.88 | 68.65 | 90.71 | 0.43 | 37.41 | 70.08 |
| ULRT4_2.88 | Archaea | 113 | 1638 | 1.32 | 47.44 | 90.70 | 0.00 | 46.70 | 85.59 |
| UT4_2.85 | Bacteria | 374 | 2542 | 2.79 | 73.37 | 90.68 | 1.28 | 46.42 | 80.29 |
| UT3_3.9 | Archaea | 73 | 1441 | 1.43 | 37.47 | 90.68 | 0.27 | 53.16 | 81.75 |
| ULRT4_2.78 | Archaea | 104 | 1806 | 1.71 | 37.73 | 90.68 | 0.00 | 46.95 | 75.75 |
| ULRT4_3.30 | Bacteria | 34 | 1672 | 1.70 | 61.96 | 90.60 | 0.00 | 70.45 | 91.57 |
| UT3_3.56 | Archaea | 15 | 1753 | 1.77 | 43.72 | 90.55 | 0.00 | 46.26 | 77.07 |
| ALRT3_1.34 | Bacteria | 447 | 4773 | 5.04 | 62.34 | 90.54 | 1.03 | 47.71 | 80.93 |
| UT3_1.74 | Bacteria | 362 | 4620 | 5.77 | 57.68 | 90.52 | 0.00 | 32.55 | 69.76 |
| ULRT3_1.91 | Bacteria | 286 | 3365 | 3.47 | 73.16 | 90.52 | 0.43 | 44.73 | 80.68 |
| UT4_1.129 | Bacteria | 319 | 2652 | 2.79 | 73.11 | 90.34 | 3.39 | 47.02 | 78.54 |
| UT4_2.71 | Bacteria | 209 | 2121 | 2.11 | 64.81 | 90.31 | 1.42 | 63.37 | 84.58 |
| UT3_3.3 | Bacteria | 209 | 3053 | 3.35 | 68.09 | 90.31 | 0.43 | 39.70 | 74.48 |
| ALRT3_2.22 | Bacteria | 489 | 4955 | 5.37 | 71.67 | 90.29 | 0.10 | 32.41 | 67.10 |
| UT4_3.74 | Bacteria | 330 | 2833 | 2.72 | 67.43 | 90.28 | 4.94 | 41.09 | 72.47 |
| ULRT4_2.116 | Bacteria | 638 | 5781 | 6.46 | 64.19 | 90.26 | 0.00 | 30.10 | 67.95 |
| UT4_1.10 | Bacteria | 16 | 2153 | 2.25 | 58.30 | 90.19 | 1.82 | 52.53 | 76.73 |
| ULRT4_3.79 | Bacteria | 323 | 3070 | 3.27 | 66.08 | 90.09 | 1.14 | 37.72 | 63.09 |
| ULRT3_2.90 | Bacteria | 220 | 4793 | 4.95 | 62.26 | 90.07 | 0.79 | 45.40 | 78.36 |
| ULRT4_1.5 | Bacteria | 331 | 2292 | 2.33 | 72.61 | 90.02 | 0.43 | 53.23 | 83.03 |
| ULRT4_3.116 | Bacteria | 15 | 2880 | 2.99 | 58.70 | 90.02 | 0.79 | 55.24 | 85.03 |
| ALRT4_2.14 | Bacteria | 2158 | 11208 | 10.41 | 66.60 | 90.01 | 2.44 | 42.52 | 78.30 |

**Table S5.** The taxonomy of the 39 genome bins which harbored genes assigned to K00117.

| **Bins** | **Phylum/Class** | **Taxonomy based on GTDBKT and Phylophlan** | **16S rRNA (> 150 bp)** | **16S rRNA identity (%)** | **Accession No. of aligned sequences** | **No. of genes associated with K00117** |
| --- | --- | --- | --- | --- | --- | --- |
|  |  |  |  |  |  |  |
| ALRT3_3.63 | Acidobacteria | o__Luteitaleales | Yes | 100 | uncultured bacterium (KF785264.1) | 11 |
| ALRT4_1.19 |  | f__Luteitaleaceae | Yes | 98.9 | Uncultured *Acidobacteria* bacterium (JX114487.1) | 7 |
| ULRT4_1.29 |  | f__Acidobacteriaceae | Yes | 99.7 | Uncultured *Acidobacterium* (HQ730655.1) | 1 |
| ULRT4_1.77 |  | g__*Terracidiphilus* |  |  |  | 2 |
| ULRT4_2.107 |  | g__*Terracidiphilus* |  |  |  | 1 |
| ULRT4_2.48 |  | f__Acidobacteriaceae |  |  |  | 2 |
| ULRT4_2.74 |  | g__*Terracidiphilus* | Yes | 98.9 | Uncultured Acidobacteriaceae bacterium (EF019731.1) | 1 |
| ULRT4_3.13 |  | g__*Terracidiphilus* |  |  |  | 1 |
| ULRT4_3.50 |  | f__Solibacteraceae |  |  |  | 5 |
| ULRT4_3.75 |  | g__*Terracidiphilus* | Yes | 100 | Uncultured bacterium (JN168353.1) | 1 |
| ULRT3_1.133 |  | g__*Silvibacterium* | Yes | 97.3 | Uncultured Acidobacteria bacterium (KM200392.1) | 1 |
| ULRT3_3.130 |  | g__*Acidobacterium* | Yes | 97.8 | Uncultured *Acidobacterium* (HQ730655.1) | 1 |
| ALRT3_1.33 | Alphaproteobacteria | g__*Bradyrhizobium* |  |  |  | 2 |
| ALRT3_3.36 |  | g__*Porphyrobacter* |  |  |  | 4 |
| ALRT4_1.43 |  | g__*Sphingomonas* | Yes | 98.2 | Uncultured *Sphingomonas* (KC138682.1) | 1 |
| ULRT4_1.51 |  | g__*Acidiphilium* |  |  |  | 1 |
| ULRT3_1.83 |  | f__Hyphomonadaceae | Yes | 97.7 | Caulobacteraceae bacterium OTSz_A_272 (CP013244.1) | 1 |
| ULRT3_2.126 |  | g__*Acidiphilium* |  |  |  | 1 |
| ULRT3_3.52 |  | g__*Acidiphilium* | Yes | 96.1 | *Acidiphilium multivorum* AIU301 (AP012035.1) | 1 |
| ALRT4_2.11 | Bacteroidetes | f__Saprospiraceae |  |  |  | 2 |
| ULRT3_1.11 |  | g__*Ohtaekwangia* |  |  |  | 1 |
| ULRT3_1.160 |  | f__Cyclobacteriaceae |  |  |  | 2 |
| ULRT3_1.60 |  | g__*Flavisolibacter* | Yes | 96.2 | uncultured *Flavisolibacter* (KC329606.1) | 1 |
| ULRT3_1.79 |  | f__Chitinophagaceae | Yes | 98.7 | Uncultured Chitinophagaceae bacterium (KT182496.1) | 1 |
| ALRT3_2.16 | Betaproteobacteria | g__*Acidovorax* |  |  |  | 1 |
| ALRT4_1.10 | Deltaproteobacteria | c__*Deltaproteobacteria* |  |  |  | 2 |
| ALRT3_1.7 | Gammaproteobacteria | f__Sinobacteraceae | Yes | 98.9 | Uncultured Sinobacteraceae bacterium (KJ192005.1) | 2 |
| ALRT3_3.35 |  | f__Sinobacteraceae | Yes | 98.9 | Uncultured Sinobacteraceae bacterium (KJ192005.1) | 2 |
| ALRT3_3.62 |  | f__Steroidobacteraceae | Yes | 98.9 | *Steroidobacter denitrificans* strain DSM 18526 (CP011971.1) | 1 |
| ALRT3_3.83 |  | f__Steroidobacteraceae |  |  |  | 1 |
| ALRT4_1.24 |  | f__Steroidobacteraceae |  |  |  | 3 |
| ALRT4_2.13 |  | c__*Gammaproteobacteria* |  |  |  | 11 |
| ALRT4_2.36 |  | f__Steroidobacteraceae |  |  |  | 2 |
| ALRT4_3.1 |  | g__*Steroidobacter* |  |  |  | 3 |
| ULRT4_3.172 |  | c__*Gammaproteobacteria* | Yes | 96.7 | Uncultured bacterium (LN567524.1) | 1 |
| ULRT3_2.14 |  | f__Rhodanobacteraceae |  |  |  | 1 |
| ULRT3_2.161 | Gemmatimonadota | f__Gemmatimonadaceae | Yes | 98.2 | Uncultured bacterium (JN869089.1) | 2 |
| ULRT3_2.4 |  | f__Gemmatimonadaceae |  |  |  | 4 |
| UT4_2.26 | Planctomycetes | f__Isosphaeraceae |  |  |  | 1 |

**Table S6.** Pearson correlations between the relative abundances of four *gcd*-containing genome bins and the concentration of bioavailable soil P.

| **Bin** | ***r*** | ***P*** value |
| --- | --- | --- |
| ALRT3_3.36 | 0.72 | 0.0008 |
| ALRT3_3.35 | 0.72 | 0.0007 |
| ALRT3_3.62 | 0.70 | 0.0011 |
| ALRT3_3.83 | 0.72 | 0.0008 |

**Table S7.** Putative mobile elements identified in the scaffolds containing *gcd* genes in the 39 genome bins.

| **Bins** | **Putative ORFs** | **eggNOG ID** | **Functional annotations** | **Identity (%)** | **Alignment_length (bp)** | **Query_start** | **Query_end** | **E-value** |
| --- | --- | --- | --- | --- | --- | --- | --- | --- |
| ALRT4_1.19 | NODE_834_length_19721_cov_3.367884_2 | COG1943 | Transposase | 38.9 | 175 | 1 | 172 | 1.40E-30 |
| ULRT4_2.74 | NODE_315_length_138466_cov_2.807205_63 | COG3857 | The heterodimer acts as both an ATP-dependent DNA helicase and an ATP-dependent, dual-direction single-stranded exonuclease. Recognizes the chi site generating a DNA molecule suitable for the initiation of homologous recombination | 34.3 | 898 | 38 | 918 | 5.40E-136 |
| ULRT4_2.74 | NODE_315_length_138466_cov_2.807205_73 | COG3677 | Transposase | 52.8 | 163 | 1 | 163 | 2.20E-44 |
| ULRT4_2.74 | NODE_315_length_138466_cov_2.807205_85 | COG3547 | Transposase (IS116 IS110 IS902 family) | 34.0 | 530 | 42 | 505 | 9.50E-66 |
| ULRT4_2.74 | NODE_315_length_138466_cov_2.807205_22 | COG0582 | viral genome integration into host DNA | 45.5 | 134 | 17 | 150 | 1.10E-22 |
| ULRT4_2.74 | NODE_315_length_138466_cov_2.807205_62 | COG1074 | ATP-dependent DNA helicase activity | 38.0 | 1230 | 12 | 1215 | 6.70E-211 |
| ULRT4_3.50 | NODE_237_length_160176_cov_7.274486_148 | COG3547 | Transposase (IS116 IS110 IS902 family) | 61.6 | 216 | 1 | 216 | 1.80E-69 |
| ULRT4_3.50 | NODE_442_length_106212_cov_7.243584_69 | COG3385 | transposase activity | 58.5 | 306 | 1 | 304 | 1.20E-95 |
| ULRT4_3.50 | NODE_65_length_326158_cov_7.227797_273 | COG0582 | viral genome integration into host DNA | 34.4 | 131 | 3 | 131 | 2.90E-09 |
| ULRT4_3.50 | NODE_156_length_206298_cov_7.274317_1 | COG2963 | transposase activity | 73.5 | 49 | 45 | 93 | 1.40E-10 |
| ULRT4_3.75 | NODE_5_length_984360_cov_4.214471_485 | COG0353 | May play a role in DNA repair. It seems to be involved in an RecBC-independent recombinational process of DNA repair. It may act with RecF and RecO | 80.2 | 192 | 1 | 192 | 7.10E-83 |
| ULRT4_3.75 | NODE_5_length_984360_cov_4.214471_778 | COG3636 | A helicase nuclease that prepares dsDNA breaks (DSB) for recombinational DNA repair. Binds to DSBs and unwinds DNA via a highly rapid and processive ATP-dependent bidirectional helicase activity. Unwinds dsDNA until it encounters a Chi (crossover hotspot instigator) sequence from the 3' direction. Cuts ssDNA a few nucleotides 3' to the Chi site. The properties and activities of the enzyme are changed at Chi. The Chi-altered holoenzyme produces a long 3'-ssDNA overhang and facilitates RecA-binding to the ssDNA for homologous DNA recombination and repair. Holoenzyme degrades any linearized DNA that is unable to undergo homologous recombination. In the holoenzyme this subunit has ssDNA-dependent ATPase and 5'-3' helicase activity. When added to pre-assembled RecBC greatly stimulates nuclease activity and augments holoenzyme processivity. Negatively regulates the RecA-loading ability of RecBCD | 50.5 | 103 | 1 | 102 | 4.00E-19 |
| ULRT4_3.75 | NODE_5_length_984360_cov_4.214471_745 | COG4584 | PFAM Integrase catalytic | 40.1 | 464 | 2 | 463 | 5.90E-94 |
| ULRT4_3.75 | NODE_5_length_984360_cov_4.214471_1 | COG1943 | Transposase | 50.6 | 154 | 1 | 154 | 1.10E-39 |
| ULRT4_3.75 | NODE_5_length_984360_cov_4.214471_259 | COG1074 | ATP-dependent DNA helicase activity | 38.3 | 1218 | 10 | 1204 | 2.60E-199 |
| ULRT4_3.75 | NODE_5_length_984360_cov_4.214471_599 | COG3636 | A helicase nuclease that prepares dsDNA breaks (DSB) for recombinational DNA repair. Binds to DSBs and unwinds DNA via a highly rapid and processive ATP-dependent bidirectional helicase activity. Unwinds dsDNA until it encounters a Chi (crossover hotspot instigator) sequence from the 3' direction. Cuts ssDNA a few nucleotides 3' to the Chi site. The properties and activities of the enzyme are changed at Chi. The Chi-altered holoenzyme produces a long 3'-ssDNA overhang and facilitates RecA-binding to the ssDNA for homologous DNA recombination and repair. Holoenzyme degrades any linearized DNA that is unable to undergo homologous recombination. In the holoenzyme this subunit has ssDNA-dependent ATPase and 5'-3' helicase activity. When added to pre-assembled RecBC greatly stimulates nuclease activity and augments holoenzyme processivity. Negatively regulates the RecA-loading ability of RecBCD | 68.1 | 94 | 1 | 94 | 1.40E-28 |
| ULRT4_3.75 | NODE_5_length_984360_cov_4.214471_359 | COG4974 | Belongs to the 'phage' integrase family | 65.8 | 322 | 17 | 337 | 1.90E-110 |
| ULRT4_3.75 | NODE_5_length_984360_cov_4.214471_246 | COG3636 | A helicase nuclease that prepares dsDNA breaks (DSB) for recombinational DNA repair. Binds to DSBs and unwinds DNA via a highly rapid and processive ATP-dependent bidirectional helicase activity. Unwinds dsDNA until it encounters a Chi (crossover hotspot instigator) sequence from the 3' direction. Cuts ssDNA a few nucleotides 3' to the Chi site. The properties and activities of the enzyme are changed at Chi. The Chi-altered holoenzyme produces a long 3'-ssDNA overhang and facilitates RecA-binding to the ssDNA for homologous DNA recombination and repair. Holoenzyme degrades any linearized DNA that is unable to undergo homologous recombination. In the holoenzyme this subunit has ssDNA-dependent ATPase and 5'-3' helicase activity. When added to pre-assembled RecBC greatly stimulates nuclease activity and augments holoenzyme processivity. Negatively regulates the RecA-loading ability of RecBCD | 83.3 | 90 | 9 | 98 | 7.90E-33 |
| ULRT4_3.75 | NODE_5_length_984360_cov_4.214471_127 | COG1200 | Critical role in recombination and DNA repair. Helps process Holliday junction intermediates to mature products by catalyzing branch migration. Has a DNA unwinding activity characteristic of a DNA helicase with a 3'- to 5'- polarity. Unwinds branched duplex DNA (Y-DNA) | 71.4 | 755 | 4 | 757 | 9.30E-304 |
| ULRT4_3.75 | NODE_5_length_984360_cov_4.214471_258 | COG3857 | The heterodimer acts as both an ATP-dependent DNA helicase and an ATP-dependent, dual-direction single-stranded exonuclease. Recognizes the chi site generating a DNA molecule suitable for the initiation of homologous recombination | 37.1 | 901 | 1 | 868 | 2.00E-132 |
| ULRT4_3.75 | NODE_5_length_984360_cov_4.214471_360 | COG4974 | Belongs to the 'phage' integrase family | 70.3 | 313 | 14 | 326 | 3.00E-118 |
| ULRT4_3.75 | NODE_5_length_984360_cov_4.214471_807 | COG4974 | Belongs to the 'phage' integrase family | 50.9 | 397 | 1 | 395 | 2.30E-104 |
| ULRT4_3.75 | NODE_5_length_984360_cov_4.214471_608 | COG0817 | Nuclease that resolves Holliday junction intermediates in genetic recombination. Cleaves the cruciform structure in supercoiled DNA by nicking to strands with the same polarity at sites symmetrically opposed at the junction in the homologous arms and leaves a 5'-terminal phosphate and a 3'-terminal hydroxyl group | 69.2 | 169 | 1 | 169 | 6.70E-53 |
| ULRT4_3.75 | NODE_5_length_984360_cov_4.214471_3 | COG1943 | Transposase | 62.5 | 80 | 1 | 80 | 6.10E-23 |
| ULRT4_3.75 | NODE_5_length_984360_cov_4.214471_664 | COG4679 | PFAM Phage derived protein Gp49-like (DUF891) | 67.7 | 96 | 1 | 96 | 5.20E-29 |
| ULRT4_3.75 | NODE_5_length_984360_cov_4.214471_281 | COG2256 | atpase related to the helicase subunit of the holliday junction resolvase | 79.1 | 426 | 21 | 446 | 7.00E-190 |
| ULRT4_3.75 | NODE_5_length_984360_cov_4.214471_595 | COG4683 | PFAM Phage derived protein Gp49-like (DUF891) | 50.4 | 113 | 1 | 113 | 1.60E-26 |
| ULRT4_3.75 | NODE_5_length_984360_cov_4.214471_21 | COG0514 | ATP-dependent DNA helicase (RecQ) | 54.1 | 706 | 2 | 701 | 1.10E-194 |
| ULRT4_3.75 | NODE_5_length_984360_cov_4.214471_800 | COG0507 | A helicase nuclease that prepares dsDNA breaks (DSB) for recombinational DNA repair. Binds to DSBs and unwinds DNA via a highly rapid and processive ATP-dependent bidirectional helicase activity. Unwinds dsDNA until it encounters a Chi (crossover hotspot instigator) sequence from the 3' direction. Cuts ssDNA a few nucleotides 3' to the Chi site. The properties and activities of the enzyme are changed at Chi. The Chi-altered holoenzyme produces a long 3'-ssDNA overhang and facilitates RecA-binding to the ssDNA for homologous DNA recombination and repair. Holoenzyme degrades any linearized DNA that is unable to undergo homologous recombination. In the holoenzyme this subunit has ssDNA-dependent ATPase and 5'-3' helicase activity. When added to pre-assembled RecBC greatly stimulates nuclease activity and augments holoenzyme processivity. Negatively regulates the RecA-loading ability of RecBCD | 71.1 | 926 | 1 | 920 | 0.00E+00 |
| ULRT4_3.75 | NODE_5_length_984360_cov_4.214471_805 | COG4634 | DNA integration | 62.6 | 139 | 3 | 140 | 1.70E-44 |
| ULRT3_1.133 | NODE_98_length_167005_cov_4.019013_7 | COG1943 | Transposase | 65.3 | 118 | 1 | 118 | 7.20E-36 |
| ULRT3_1.133 | NODE_98_length_167005_cov_4.019013_6 | COG3547 | Transposase (IS116 IS110 IS902 family) | 70.8 | 339 | 1 | 338 | 2.10E-133 |
| ULRT3_1.133 | NODE_98_length_167005_cov_4.019013_54 | COG0632 | Four-way junction helicase activity | 80.1 | 196 | 1 | 196 | 9.10E-78 |
| ULRT3_3.130 | NODE_90_length_155112_cov_7.798458_79 | COG2256 | Atpase related to the helicase subunit of the holliday junction resolvase | 78.7 | 445 | 6 | 446 | 1.20E-194 |
| ALRT3_1.33 | NODE_752_length_26441_cov_7.394013_8 | COG1381 | Involved in DNA repair and RecF pathway recombination | 93.6 | 250 | 1 | 250 | 9.10E-131 |
| ALRT3_1.33 | NODE_752_length_26441_cov_7.394013_7 | COG0188 | A type II topoisomerase that negatively supercoils closed circular double-stranded (ds) DNA in an ATP-dependent manner to modulate DNA topology and maintain chromosomes in an underwound state. Negative supercoiling favors strand separation, and DNA replication, transcription, recombination and repair, all of which involve strand separation. Also able to catalyze the interconversion of other topological isomers of dsDNA rings, including catenanes and knotted rings. Type II topoisomerases break and join 2 DNA strands simultaneously in an ATP-dependent manner | 92.9 | 751 | 1 | 751 | 0.00E+00 |
| ALRT3_3.36 | NODE_71_length_170708_cov_7.744858_47 | COG4581 | Dead DEAH box helicase | 66.7 | 835 | 20 | 849 | 4.10E-308 |
| ALRT3_3.36 | NODE_176_length_99990_cov_7.107476_80 | COG4240 | Helicase activity | 55.0 | 278 | 5 | 278 | 4.20E-76 |
| ALRT3_3.36 | NODE_176_length_99990_cov_7.107476_15 | 33AZ2 | Phage shock protein B | 70.7 | 99 | 1 | 99 | 6.00E-33 |
| ALRT3_3.36 | NODE_176_length_99990_cov_7.107476_14 | COG1842 | Phage shock protein A | 80.0 | 275 | 20 | 279 | 4.20E-108 |
| ULRT4_1.51 | NODE_1373_length_39745_cov_2.830902_28 | COG2256 | Atpase related to the helicase subunit of the holliday junction resolvase | 75.9 | 449 | 5 | 453 | 1.50E-184 |
| ULRT3_2.126 | NODE_20_length_409521_cov_25.980811_380 | COG3293 | Transposase | 76.1 | 213 | 1 | 213 | 5.70E-89 |
| ULRT3_2.126 | NODE_20_length_409521_cov_25.980811_206 | COG2256 | Atpase related to the helicase subunit of the holliday junction resolvase | 87.0 | 416 | 1 | 416 | 7.80E-204 |
| ULRT3_2.126 | NODE_20_length_409521_cov_25.980811_383 | COG4974 | Belongs to the 'phage' integrase family | 63.8 | 307 | 23 | 327 | 2.30E-97 |
| ULRT3_2.126 | NODE_20_length_409521_cov_25.980811_71 | COG1381 | Involved in DNA repair and RecF pathway recombination | 78.5 | 247 | 1 | 247 | 5.00E-105 |
| ULRT3_2.126 | NODE_20_length_409521_cov_25.980811_72 | COG0188 | A type II topoisomerase that negatively supercoils closed circular double-stranded (ds) DNA in an ATP-dependent manner to modulate DNA topology and maintain chromosomes in an underwound state. Negative supercoiling favors strand separation, and DNA replication, transcription, recombination and repair, all of which involve strand separation. Also able to catalyze the interconversion of other topological isomers of dsDNA rings, including catenanes and knotted rings. Type II topoisomerases break and join 2 DNA strands simultaneously in an ATP-dependent manner | 87.1 | 745 | 1 | 745 | 0.00E+00 |
| ULRT3_2.126 | NODE_20_length_409521_cov_25.980811_122 | COG3378 | Phage plasmid primase P4 family | 47.2 | 316 | 7 | 322 | 1.00E-60 |
| ULRT3_2.126 | NODE_20_length_409521_cov_25.980811_126 | COG0582 | Viral genome integration into host DNA | 38.9 | 437 | 4 | 422 | 1.50E-77 |
| ULRT3_2.126 | NODE_20_length_409521_cov_25.980811_101 | COG0188 | A type II topoisomerase that negatively supercoils closed circular double-stranded (ds) DNA in an ATP-dependent manner to modulate DNA topology and maintain chromosomes in an underwound state. Negative supercoiling favors strand separation, and DNA replication, transcription, recombination and repair, all of which involve strand separation. Also able to catalyze the interconversion of other topological isomers of dsDNA rings, including catenanes and knotted rings. Type II topoisomerases break and join 2 DNA strands simultaneously in an ATP-dependent manner | 89.5 | 904 | 1 | 904 | 0.00E+00 |
| ULRT3_2.126 | NODE_20_length_409521_cov_25.980811_36 | COG0353 | May play a role in DNA repair. It seems to be involved in an RecBC-independent recombinational process of DNA repair. It may act with RecF and RecO | 82.5 | 194 | 2 | 195 | 4.30E-83 |
| ULRT3_2.126 | NODE_20_length_409521_cov_25.980811_352 | COG1200 | Critical role in recombination and DNA repair. Helps process Holliday junction intermediates to mature products by catalyzing branch migration. Has a DNA unwinding activity characteristic of a DNA helicase with a 3'- to 5'- polarity. Unwinds branched duplex DNA (Y-DNA) | 79.0 | 687 | 1 | 687 | 2.60E-305 |
| ULRT3_2.126 | NODE_20_length_409521_cov_25.980811_88 | COG4974 | Belongs to the 'phage' integrase family | 75.1 | 317 | 1 | 316 | 6.80E-123 |
| ULRT3_2.126 | NODE_20_length_409521_cov_25.980811_384 | COG1203 | CRISPR-associated helicase, Cas3 | 45.0 | 825 | 5 | 803 | 1.70E-165 |
| ULRT3_3.52 | NODE_610_length_54885_cov_23.596498_22 | COG0188 | A type II topoisomerase that negatively supercoils closed circular double-stranded (ds) DNA in an ATP-dependent manner to modulate DNA topology and maintain chromosomes in an underwound state. Negative supercoiling favors strand separation, and DNA replication, transcription, recombination and repair, all of which involve strand separation. Also able to catalyze the interconversion of other topological isomers of dsDNA rings, including catenanes and knotted rings. Type II topoisomerases break and join 2 DNA strands simultaneously in an ATP-dependent manner | 75.4 | 886 | 1 | 886 | 0.00E+00 |
| ULRT3_1.11 | NODE_173_length_121105_cov_4.463046_30 | COG0497 | DNA recombination | 48.3 | 549 | 1 | 549 | 5.20E-139 |
| ULRT3_1.160 | NODE_440_length_69564_cov_4.027676_5 | COG0582 | Viral genome integration into host DNA | 34.7 | 412 | 3 | 394 | 7.90E-52 |
| ULRT3_1.79 | NODE_16_length_375232_cov_4.606830_105 | COG3547 | Transposase (IS116 IS110 IS902 family) | 76.1 | 322 | 1 | 322 | 3.50E-143 |
| ULRT3_1.79 | NODE_16_length_375232_cov_4.606830_51 | COG0497 | DNA recombination | 65.6 | 552 | 1 | 552 | 5.90E-199 |
| ULRT3_1.79 | NODE_16_length_375232_cov_4.606830_28 | COG1200 | Critical role in recombination and DNA repair. Helps process Holliday junction intermediates to mature products by catalyzing branch migration. Has a DNA unwinding activity characteristic of a DNA helicase with a 3'- to 5'- polarity. Unwinds branched duplex DNA (Y-DNA) | 69.3 | 696 | 14 | 709 | 1.40E-285 |
| ALRT4_1.24 | NODE_29_length_108450_cov_6.258905_14 | COG1199 | ATP-dependent helicase activity | 52.6 | 612 | 1 | 603 | 1.50E-161 |
| ALRT4_2.13 | NODE_189_length_46937_cov_3.298014_22 | COG0513 | Belongs to the DEAD box helicase family | 58.8 | 585 | 13 | 597 | 1.00E-178 |
| ALRT4_2.13 | NODE_382_length_27566_cov_2.977340_1 | COG0514 | ATP-dependent DNA helicase (RecQ) | 65.3 | 360 | 2 | 358 | 7.70E-134 |
| ALRT4_2.13 | NODE_289_length_34138_cov_3.512772_15 | COG0513 | Belongs to the DEAD box helicase family | 66.7 | 390 | 1 | 389 | 1.10E-136 |
| ALRT4_2.13 | NODE_473_length_23578_cov_3.276544_8 | COG0582 | Viral genome integration into host DNA | 51.2 | 209 | 1 | 207 | 4.90E-54 |
| ALRT4_3.1 | NODE_92_length_44552_cov_2.772432_32 | COG0513 | Belongs to the DEAD box helicase family | 65.9 | 384 | 1 | 374 | 4.90E-140 |
| ULRT4_3.172 | NODE_4_length_1012241_cov_3.751626_35 | COG0582 | Viral genome integration into host DNA | 27.6 | 655 | 32 | 663 | 7.30E-50 |
| ULRT4_3.172 | NODE_4_length_1012241_cov_3.751626_371 | COG1200 | Critical role in recombination and DNA repair. Helps process Holliday junction intermediates to mature products by catalyzing branch migration. Has a DNA unwinding activity characteristic of a DNA helicase with a 3'- to 5'- polarity. Unwinds branched duplex DNA (Y-DNA) | 63.1 | 686 | 19 | 704 | 1.00E-243 |
| ULRT4_3.172 | NODE_4_length_1012241_cov_3.751626_320 | COG2433 | Pfam Transposase IS66 | 39.4 | 221 | 8 | 222 | 1.60E-17 |
| ULRT4_3.172 | NODE_4_length_1012241_cov_3.751626_452 | COG1198 | Involved in the restart of stalled replication forks. Recognizes and binds the arrested nascent DNA chain at stalled replication forks. It can open the DNA duplex, via its helicase activity, and promote assembly of the primosome and loading of the major replicative helicase DnaB onto DNA | 56.2 | 729 | 8 | 732 | 1.20E-218 |
| ULRT4_3.172 | NODE_4_length_1012241_cov_3.751626_863 | COG3677 | Transposase | 26.5 | 313 | 15 | 317 | 9.40E-20 |
| ULRT4_3.172 | NODE_4_length_1012241_cov_3.751626_162 | COG0513 | Belongs to the DEAD box helicase family | 64.8 | 415 | 12 | 426 | 2.00E-149 |
| ULRT4_3.172 | NODE_4_length_1012241_cov_3.751626_37 | COG4974 | Belongs to the 'phage' integrase family | 26.8 | 422 | 25 | 420 | 1.10E-22 |
| ULRT4_3.172 | NODE_4_length_1012241_cov_3.751626_585 | COG0210 | DNA helicase | 65.2 | 718 | 1 | 716 | 1.20E-271 |
| ULRT4_3.172 | NODE_4_length_1012241_cov_3.751626_864 | COG2963 | Transposase activity | 25.3 | 572 | 19 | 579 | 1.40E-39 |
| ULRT4_3.172 | NODE_4_length_1012241_cov_3.751626_738 | COG4973 | Belongs to the 'phage' integrase family. XerC subfamily | 61.4 | 295 | 7 | 301 | 3.30E-95 |
| ULRT4_3.172 | NODE_4_length_1012241_cov_3.751626_866 | COG2801 | Transposase and inactivated derivatives | 29.7 | 634 | 84 | 698 | 8.70E-60 |
| ULRT4_3.172 | NODE_4_length_1012241_cov_3.751626_230 | COG0513 | Belongs to the DEAD box helicase family | 71.1 | 575 | 4 | 575 | 8.20E-215 |
| ULRT4_3.172 | NODE_4_length_1012241_cov_3.751626_649 | COG0210 | DNA helicase | 62.3 | 645 | 11 | 653 | 2.60E-228 |
| ULRT3_2.14 | NODE_439_length_92774_cov_7.394854_73 | COG0497 | DNA recombination | 80.0 | 555 | 1 | 555 | 1.00E-243 |
| ULRT3_2.14 | NODE_439_length_92774_cov_7.394854_84 | COG4584 | PFAM Integrase catalytic | 77.3 | 75 | 10 | 84 | 4.10E-24 |
| ULRT3_2.161 | NODE_281_length_117650_cov_5.484392_61 | COG1198 | Involved in the restart of stalled replication forks. Recognizes and binds the arrested nascent DNA chain at stalled replication forks. It can open the DNA duplex, via its helicase activity, and promote assembly of the primosome and loading of the major replicative helicase DnaB onto DNA | 63.7 | 763 | 7 | 753 | 9.30E-264 |
| UT4_2.26 | k127_45562_6 | COG5464 | transposase or invertase | 32.1 | 321 | 1 | 316 | 8.70E-33 |
| UT4_2.26 | k127_45562_87 | COG4634 | DNA integration | 47.8 | 115 | 1 | 115 | 4.70E-24 |
| UT4_2.26 | k127_45562_97 | COG0582 | Viral genome integration into host DNA | 34.6 | 384 | 2 | 375 | 9.20E-48 |


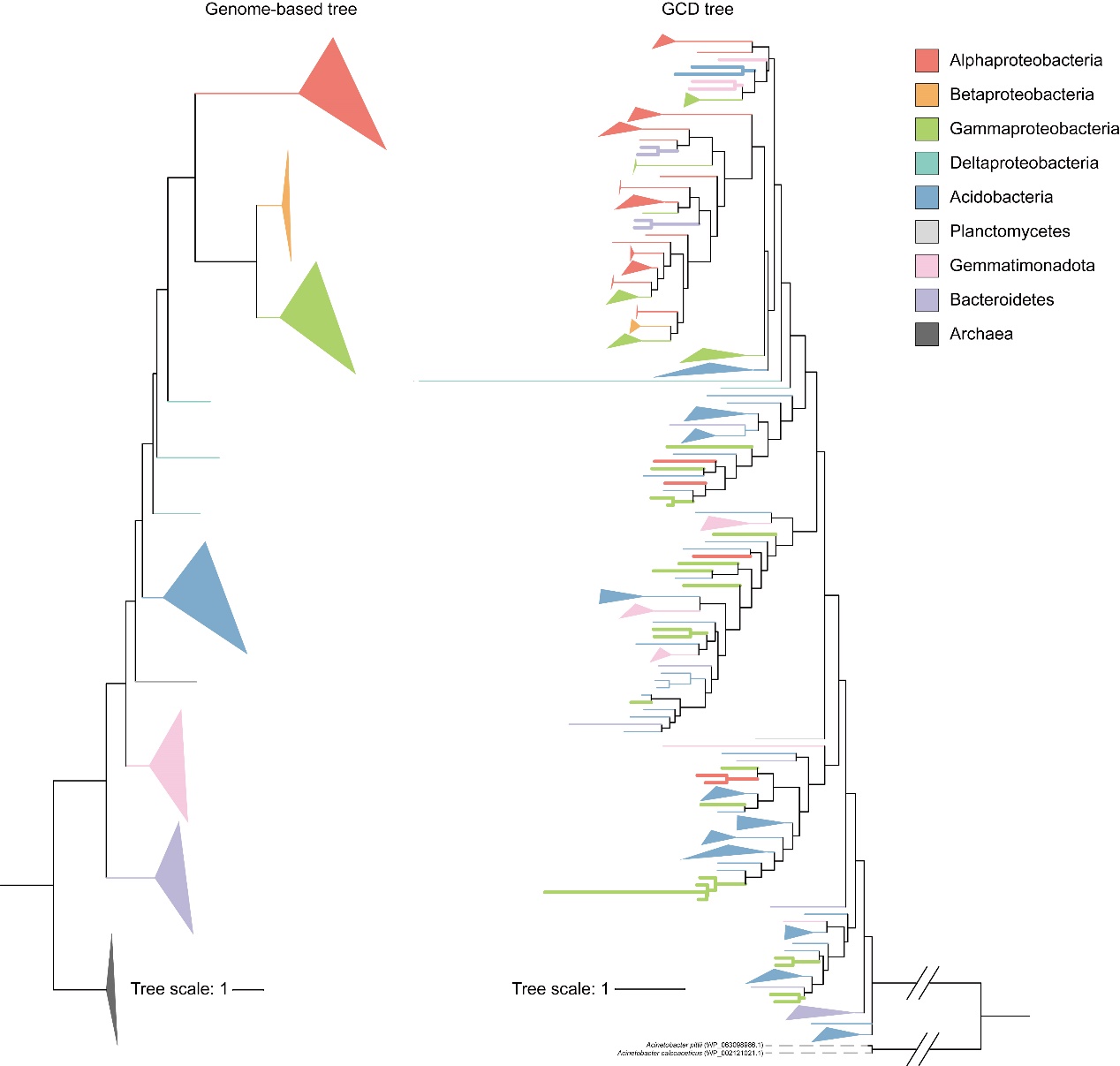


**Figure S1.** **Comparison of genome-based phylogenetic tree and GCD tree for potential phosphate-solubilizing microorganisms.** In combination with the 39 *gcd*-containing bins recovered in this study, 44 other *gcd*-harboring genomes affiliated to the same phyla as those covering by our bins were downloaded from NCBI GenBank for the construction of the trees (please refer to main text for more details). Sequences are grouped at the phylum level. Each phylum is colored differently to identify horizontal gene transfer (HGT) based on inconsistent branching patterns. The branches in bold indicate possible HGT events of *gcd* genes associated with specific phylum. Two GCD sequences identified in genomes from the class Deltaproteobacteria (*Desulfurivibrio alkaliphilus* AHT 2 and *Sorangium cellulosum* So ce56) are not shown in the GCD tree, as they are soluble GCDs.
